# Characterization of bovine uterine fluid extracellular vesicles proteomic profiles at follicular and luteal phases of the oestrous cycle

**DOI:** 10.1007/s11259-022-10052-3

**Published:** 2022-12-22

**Authors:** Johanna Piibor, Keerthie Dissanayake, Getnet Midekessa, Aneta Andronowska, Ants Kavak, Andres Waldmann, Alireza Fazeli

**Affiliations:** 1 Institute of Veterinary Medicine and Animal Sciences, Estonian University of Life Sciences, Kreutzwaldi 62, 51006 Tartu, Estonia; 2 Department of Pathophysiology, Institute of Biomedicine and Translational Medicine, University of Tartu, Ravila St. 14B, 50411 Tartu, Estonia; 3 Department of Anatomy, Faculty of Medicine, University of Peradeniva, Peradeniva, 20400 Sri Lanka; 4 Institute of Animal Reproduction and Food Research, Polish Academy of Sciences, Juliana Tuwima 10, Olsztyn, Poland; 5 Academic Unit of Reproductive and Developmental Medicine, Department of Oncology and Metabolism, Medical School, University of Sheffield, Sheffield, S10 2TN UK

**Keywords:** Extracellular vesicles, Bovine uterine fluid, Oestrous cycle, Proteomics, Fluorescence NTA

## Abstract

Extracellular vesicles (EV) have been identified in uterine fluid (UF), however the bovine UF-EV profile during different phases of the oestrous cycle has not yet been established. Therefore, we compared the UF-EV, and their protein profile at follicular and luteal phases of the oestrous cycle. UF samples were collected from healthy uteri of six live and six slaughtered cows at follicular or luteal phases. Isolation of EV was performed using tangential flow filtration followed by size exclusion chromatography. EV were characterized by nanoparticle tracking analysis (NTA), fluorescence NTA, zeta potential, and transmission electron microscopy. Mass-spectrometry was used to evaluate EV protein profile from live cows. Particle concentrations (mean ± SD) were higher (*P* < 0.05) at follicular than at luteal phase in both live (1.01 × 10^8^ ± 1.66 × 10^7^ vs 7.56 × 10^7^ ± 1.80 × 10^7^, respectively) and slaughtered cows (1.17 × 10^8^ ± 2.34 × 10^7^ vs 9.12 × 10^7^ ± 9.77 × 10^6^, respectively). The proportion of fluorescently labelled EV varied significantly between follicular and luteal phases across live (28.9 ± 1.9% vs 19.3 ± 2.8%, respectively) and slaughtered cows (26.5 ± 6.3% vs 27.3 ± 2 .7%, respectively). In total, 41 EV proteins were differentially expressed between the phases. Some of the proteins were involved in reproductive processes, cell adhesion and proliferation, and cellular metabolic processes. The results indicated differences in bovine UF-EV concentration and protein profile at follicular and luteal phases, which would suggest that EV modulate uterine microenvironment across the oestrous cycle. Further research is needed to understand the effect of EV changes throughout the oestrous cycle.

**Supplementary Information:**

The online version contains supplementary material available at 10.1007/s11259-022-10052-3.

## Background

Bovine oestrous cycle is a highly dynamic process that generally lasts 18—24 days and is characterized by two discrete phases: follicular and luteal phases. The luteal phase (generally 14—18 days) is a period in the oestrous cycle after ovulation when the *corpus luteum* (CL) is formed, while the follicular phase (generally 4—6 days) is the period from the demise of a functional CL until ovulation (Forde et al. 2011). Across these phases of the oestrous cycle, spatiotemporal changes occur in the endometrium and its secretions which are orchestrated by ovarian hormones such as oestrogen and progesterone as well as by the conceptus, if there is any (Chae et al. 2011; Faulkner et al. 2013; Martins et al. 2018). There are four main sources that contribute and influence the composition of the uterine fluid (UF), i.e., uterine glands, uterine epithelial cells, uterine vasculature and conceptus (if there is any) (Simintiras and Forde 2017). As a result of these contributions, the UF composition ultimately determines the uterine microenvironment. Several studies have described the components of the soluble secretome in UF, such as amino acids, carbohydrates, proteins, and lipids (O’Neil and Spencer 2021; Simintiras et al. 2019). These molecules in UF provide a microenvironment for growth and survival of pre-implantation embryo and mediate embryo-maternal communication (Spencer 2014). While optimum communication and nurture of preimplantation embryos mediated by UF leads to improved embryonic implantation and development, deranged or suboptimum conditions can lead to implantation failure and pregnancy loss. A meta-analysis has shown that ~ 28% of the embryos do not survive and develop beyond 7 days of gestation in beef cattle (Reese et al. 2020). Improved understanding of the uterine microenvironment and UF composition would help to develop preventive measures for such losses.

Generally, intercellular communication has been believed to be mediated by different secretory soluble factors such as cytokines and hormones. However, recent studies have discovered the role of extracellular vesicles (EV) in cell-to-cell communication (Burns et al. 2018; Kusama et al. 2018). EV are lipid membrane-bound nanoparticles, that contain different biomolecules such as proteins, nucleic acids, lipids, genomic DNA, mRNA, and miRNAs. These nano-sized particles are produced by cells and secreted to biofluids such as follicular (Pioltine et al. 2020), amniotic (Lange-Consiglio et al. 2020), oviductal (Hamdi et al. 2021), and uterine fluid (Hamdi et al. 2021; Nakamura et al. 2019), where they are up taken by the cells. EV containing biomolecules that are transported from one cell or tissue to another, exert biological roles and influence several normal physiological and pathological conditions (Hamdi et al. 2021; Simon et al. 2018).

During both phases of the oestrous cycle considerable intercellular communications occur in the uterine milieu that are essential to uterine pathophysiology, implantation, and pregnancy establishment. Increasing evidence shows that EV regulate different reproductive events such as sperm/ovum maturation (Cabarello et al. 2010; Pioltine et al. 2020; Sullivan et al. 2005), coordination of capacitation/acrosome reaction (Hasan et al. 2021; Burns et al. 2014), endometrial-embryo crosstalk (Burns et al. 2014; Dissanayake et al. 2021b; Es-Haghi et al. 2019) and regulation of maternal immune system allowing the conceptus attachment to the endometrial epithelium (Nakamura et al. 2019). Ovine UF-EV have been internalized by ovine trophectodermal cells in vitro giving rise to enhanced proliferation and the secretion of interferon-τ, the pregnancy recognition molecule of ruminant pregnancy (Ruiz-González et al. 2015). Nakamura et al. (2019) supplemented bovine endometrial epithelial cells (EEC) with UF-EV isolated from day 17 or 20 of pregnancy. EEC-EV released in response to such supplementation were observed to down-regulate immune system related transcripts of the day 20 UF-EV supplemented group compared to that of day 17. This may partially illustrate how the EV can modulate the maternal immune response in the uterine milieu facilitating implantation (Nakamura et al. 2019). Even after implantation, the secretion of EV by endometrium continues throughout pregnancy, which means that EV role in reproduction is not only limited to the establishment of the pregnancy, but it continues well beyond (Tannetta et al. 2014).

The potential role of EV as a ‘liquid biopsy’ is also well studied topic (Schobers et al. 2021). UF-EV may contain useful markers for evaluating uterine endometrial status (Andrade et al. 2019; Herrero et al. 2019). While UF can be directly analysed to measure certain soluble biomolecules such as proteins, these methods cannot distinguish biomolecules packaged in EV from those that are soluble in the UF matrix. Therefore, characterization of UF-EV (a less studied components of UF and distinct from the soluble secretome) during the oestrous cycle in cattle may yield to new biomarkers for evaluation of the uterine status. To date, only miRNAs have been studied from bovine UF-EV across the oestrous cycle (Hamdi et al. 2021). Therefore, the objective of the present study was to isolate and characterize bovine UF-EV, and to compare their differences based on physical characteristics and proteomic profiles during follicular and luteal phases of the oestrous cycle. In addition, we compared the physical characteristics of UF-EV of samples obtained from live animals or those immediately after slaughter (2 – 3 h). Such information may lead to a reduction in the use of live animals in future experiments and will strengthen the implementation of the 3Rs (replacement, reduction, and refinement) principles in experiments using animals.

## Materials and methods

All experiments involving animals were approved by the Committee for Conducting Animal Experiments at the Ministry of Rural Affairs, Estonia (Approval number 200 from 09.07.2021).

### Experimental design

**Fig. 1:**
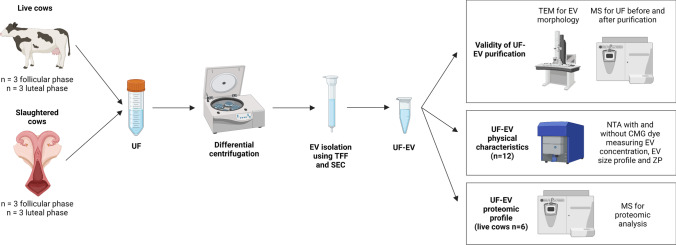
**Experimental design.** Uterine fluid (UF) samples were collected from 6 live and 6 slaughtered cows from which 3 were at follicular and 3 luteal phases. Next, UF samples were subjected to differential centrifugation and stored at -80 °C. The samples were thawed one by one and extracellular vesicles (EV) were isolated using tangential flow filtration (TFF) and size exclusion chromatography (SEC) method. The validity of UF-EV purification was performed using transmission electron microscopy (TEM) for EV morphology, and mass-spectrometry (MS) with samples before and after purification to evaluate the enrichment of EV protein markers in the samples. Next, UF-EV physical characteristics were evaluated in slaughtered and live cows at follicular and luteal phases by fluorescence nanoparticle tracking analysis (NTA) using CellMask™ Green Plasma Membrane (CMG) stain measuring EV concentration, EV size profile and zeta potential (ZP). Furthermore, UF-EV proteomic profile was evaluated in live cows at follicular and luteal phases using MS. Created with BioRender.com

Three experiments were carried out to accomplish the objectives (Fig. 1). In the first experiment, the success in the isolation of EV from UF samples was determined using differential centrifugation, tangential flow filtration (TFF) and size exclusion chromatography (SEC) procedures as described in the current investigation. UF-EV samples were subjected to transmission electron microscopy (TEM) to visualize the presence of lipid bilayer membranous vesicles. Furthermore, an UF sample before and after UF-EV isolation were analysed using mass-spectrometry (MS) proteomic analysis. Thereafter, the enrichment of different EV proteomic markers and other non-EV protein markers as described by the International Society for Extracellular Vesicles (ISEV) (Théry et al. 2018) for EV proteomic analysis was investigated.

In the second experiment, UF-EV physical characteristics such as zeta potential (ZP), fluorescently labelled EV concentrations and size in different phases of the oestrous cycle from live (n = 3 in follicular phase, n = 3 in luteal phase) and slaughtered cows (n = 3 in follicular phase, n = 3 in luteal phase) were compared using fluorescence nanoparticle tracking analysis (NTA) with and without CellMask™ Green Plasma Membrane Stain (CMG).

In the third experiment, the differences in proteomic profiles of UF-EV in different phases of the oestrous cycle were determined. UF-EV proteomic profiles of 3 live cows at follicular and 3 different live cows at luteal phases of the oestrous cycle were established using MS analysis as described below.

### Sample Collection from live cows

#### Selection of cattle and evaluation of the phase of the oestrous cycle

Multiparous Holstein cows, which were clinically healthy at the day of sample collection, were used to collect UF. Cows were excluded from the study who suffered any clinical or subclinical endometritis, mastitis, clinical ketosis, strong lameness, poor body condition or had a drop in milk production in the last week before UF sampling. Evaluation of the uteri for inflammatory condition is described in Section 2.5.

Before obtaining UF, ultrasound of ovarian structures and qualitative progesterone measurements from milk were performed to confirm luteolysis, ovulation and formation of active CL. On the basis of the obtained information from ultrasound and milk progesterone measurements different phases of the oestrous cycle were determined (Supplementary file 1). Briefly, the ultrasonography was performed transrectally to evaluate the ovarian structures and to exclude cows with ovarian pathologies, such as follicular or luteal cysts (DesCôteaux et al. 2009). Progesterone in milk was measured using a qualitative milk progesterone test P4 Rapid® (Ridgeway Research Ltd, St Briavels, UK), which is a reliable method for distinguishing between low and high milk progesterone levels (Waldmann and Raud 2016). P4 Rapid® was performed such as described in the manufacturers’ instructions and the results were evaluated according to Waldmann and Raud (2016). Cows were confirmed to be in the follicular phase when the progesterone in milk was low (< 2.3 ng/mL) according to the P4 Rapid® test and no CL or ovarian cysts were detected with ultrasonographic investigation. Bovine luteal phase was confirmed, when progesterone in milk was high (> 6.9 ng/mL) according to the P4 Rapid® test and CL was detected during ultrasonographic investigations, while no ovarian cysts could be visualized.

#### UF collection

UF samples were taken from cows under low sacral epidural anaesthesia with xylazine (0.05 mg/kg) diluted in 5 mL of saline. Each uterine horn was flushed with 50 mL of PBS separately (Dulbecco’s Phosphate Buffered Saline, Sigma-Aldrich Chemie GmbH, Germany) using Foley embryo transfer catheter CH18 (Minitüb GmbH, Tiefenbach, Germany). The pooled UF (66.3 ± 12.4 mL) of both horns was recovered in a plastic tube as much as possible and transported on ice for processing in 2 to 3 h after collection. In total, three follicular and three luteal phase UF samples from live cows were collected.

### Sample collection from slaughtered cows

#### Selection of uteri and evaluation of the phase of the oestrous cycle

Clinically healthy multiparous Holstein cow uteri acquired from a slaughterhouse were used to collect UF. Cow uteri were excluded from the study who suffered clinical or subclinical endometritis and had follicular or luteal cysts. Evaluation of the uteri for inflammatory condition is described in Section 2.5. Selected uteri were transported on ice in 2 to 3 h after slaughter for evaluation of the phase of the oestrous cycle and collection of UF.

For slaughtered cows, the phase of the oestrous cycle was determined based on the morphological evaluation of the ovarian structures similarly described by Arosh et al. (2002) (Supplementary file 1). Briefly, the ovarian structures were visually evaluated (e.g. external colour, vascularization) and the sizes of CL (diameter, protuberance from ovarian surface) and follicles (diameter) in ovaries were measured (Supplementary file 2). Next, the CL was dissected, and internal characteristics of CL, such as internal colour and margins between CL and ovarian stroma were evaluated (Supplementary file 2). Cows at the time of the slaughter were considered to be at the follicular phase of the cycle, if the colour of CL was bloody or yellow to white and vascularization of CL was regressing or not visible, or ovaries did not contain a visible CL. Cows at the time of slaughter were considered to be in the luteal phase of the oestrous cycle, if the CL had a distinct margin between ovarian stroma and was coloured brown or orange with a protuberance > 1 mm outside the ovarian surface.

#### UF collection

UF samples were taken from both horns, which were flushed with 50 mL of PBS each using insemination pipette (Minitüb GmbH, Tiefenbach, Germany). The pooled UF of both horns (69.3 ± 12.1 mL) were recovered in a plastic tube as much as possible and transported on ice for further analysis in 30 min after collection. In total, three follicular and three luteal phase UF samples from slaughtered cows were collected.

### Differential centrifugation of UF samples

After collection of the UF samples from live and slaughterhouse obtained material, differential centrifugation was carried out to remove cells, cell debris, apoptotic bodies, and other impurities. Initially, the samples were centrifuged at 250 g for 5 min at 4 °C to remove cells. Next, the supernatant was transferred to another fresh tube and centrifuged at 2000 g for 10 min at 4 °C to remove cell debris. Then again, the supernatant was collected to another fresh tube and centrifuged at 10,000 g for 30 min at 4 °C to remove other impurities. The final supernatant was collected and stored at -80 °C until EV isolation.

### Evaluation of inflammatory condition of uteri

The cell pellet after the first centrifugation was collected and used to evaluate the inflammatory status of the uterus. The cytology slides were prepared, stained, and counted according to Valdmann et al. (2018). Briefly, two cytological examination slides were prepared from the cell pellet and immediately fixed with a hair dryer. The slides were stained using May-Grünwald-Giemsa staining procedure. First, cytological examination slides were placed in May-Grünwald stain (VWR Prolabo Chemicals, Leuven, Belgium) for 5 min, then transferred into diluted Giemsa stain (VWR Prolabo Chemicals, Leuven, Belgium) for 25 min and washed with distilled water. Slides were observed under light microscope under magnification of 400x and 1000x. The number of epithelial cells and polymorphonuclear neutrophils (PMN) on each slide were counted out of 100 cells. The average percentage of PMN of the two slides was used. Only UF samples, which had less than 1% of PMN were included in the investigations.

### UF-EV isolation

Using TFF methodology, UF samples were concentrated while removing small proteins and other molecules from the sample. Briefly, two syringes, one containing the UF sample were pushed gently up and down to concentrate and purify EV through TFF Easy® filtration unit (HansaBioMed Life Sciences, Tallinn, Estonia). The process was repeated until the sample volume reached to 1 mL, which was then further concentrated up to 500 µL using Amicon® Ultra-2 mL centrifugal filters (10 kDa cut-off, Merck Millipore Ltd, Darmstadt, Germany) by centrifuging at 2000 g.

EV were isolated from the purified and concentrated UF samples using a SEC method (Reshi et al. 2021). SEC columns were prepared by packing Econo-Pac® Chromatography columns (Bio-Rad, Hercules, CA USA, cat:7321010) with size exclusion chromatography resin (catalog. Sepharose 4 fast flow™, Cytvia, Uppsala, Sweden). In brief, the packed SEC columns were positioned vertically in a holder and washed by running through ultrapure Milli-Q® water (machine type: 08.2205, TKA Wasseraufbereitungssysteme GmbH, Niederelbert, Germany) and equilibrated with PBS. Then, the sample (500 µL of concentrated UF) was placed on the top of the filter of the column and immediately 20 fractions of 500 µL were collected.

#### Identification of EV fractions

The number of particles in each fraction was determined using NTA. Briefly, particle concentrations in each of the fractions were measured with NTA—ZetaView® (PMX 110 V3.0 instrument by Particle Metrix GmbH, Inning am Ammersee, Germany) coupled with ZetaView NTA software for data analysis (Dissanayake et al. 2021a). Operating instructions of the manufacturer were followed. For the auto-alignment of the instrument, 100 nm polystyrene particle size standards (Applied Microspheres B.V., Leusden, Netherlands, Catalogue no. 10100) were used. Samples were measured in the scatter mode for their total particle concentration and size profiles using the following settings: camera sensitivity 85, shutter 70, frame rate 30 frames per second (fps), number of cycles 3 and number of frames 11. Based on detectable quantities of nanoparticles during NTA measurements, the fractions with UF-EV were pooled per sample and further concentrated to 500 µL using Amicon® Ultra-2 mL centrifugal filters (Merck Millipore Ltd, Darmstadt, Germany, 10 KDs cut-off) at 2000 g for the subsequent characterization steps.

#### Protein content quantitation

Bradford Assay was used to determine the protein concentration of each fraction of the sample. First, dilutions of bovine serum albumin (BSA) standard solution (2 mg/mL, Sigma-Aldrich, USA) were prepared (1.4, 1.0, 0.5, 0.25 and 0.125 mg/mL). For negative control PBS was used. Next, three replicates of each sample, standards, and negative control of 5 µL were placed in a 96 well microplate. On top of the samples, 95 µL of Bradford Reagent (Sigma-Aldrich, St Louis, MO, USA) was added. Microplate was covered with foil and placed on a shaker for 30 s for homogenous mixing. Then the plate was incubated at room temperature (RT) for 15–30 min. Finally, the absorbance of the standards and the samples were measured with a spectrophotometer (Ledetect 96 Microplate Reader; Biomed Dr. Wieser GmbH, Salzburg, Austria) at 620 nm wavelength and the protein concentrations of each of the fractions were calculated.

### Fluorescent nanoparticle tracking analysis

#### Fluorescence labelling of UF-EV

The pooled and concentrated EV fractions were diluted using PBS to reach a particle concentration of ~ 1 × 10^10^ particles/mL. CMG stock solution (Thermo Fisher Scientific, Waltham, MA, USA, catalogue no. C37608) was prepared in 1:50 dilution with PBS. This solution was regarded as CMG stock solution. Then, 1 µL of the CMG stock solution was added to 9 µL of the samples and incubated at RT for 1 h in dark. The incubated samples were added to 990 µL of PBS to reach a final volume of 1 mL with a pH value of 7.2. The diluted EV samples (without dye) and samples with CMG dye were used for measurement of fluorescently stained particles with fluorescence NTA.

#### Fluorescence NTA and zeta potential measurements

Fluorescence NTA particle size and concentration as well as ZP measurements were performed on pooled EV fractions. Measurements were conducted using a ZetaView PMX 120 V4.1 instrument (Particle Metrix GmbH, Inning am Ammersee, Germany), while the data was analysed with ZetaView NTA software. Autoalignment of the machine was performed according to the manufacturer’s instructions as described above. The samples were measured in the scatter mode (EV only), while the samples with CMG dye (fluorescently labelled-EV) were measured in scatter (total-NP) and fluorescent mode (FL-NP). The instrument settings in the scatter mode were: sensitivity 72, shutter 100, minimum brightness 30, frame rate 30 fps, number of cycles 3 and number of frames 11. When the samples were measured in the fluorescence mode, the instrument settings used were: sensitivity 90, shutter 100, minimum brightness 25, frame rate 30 fps and number of frames 2. The ZP was measured three times in the scatter and fluorescent modes with the same measurement settings described above.

### Transmission electron microscopy

The morphology based physical characterization of EV was evaluated using TEM. In brief, 20 µL droplets of purified UF-EV from each pooled group was placed on formvar/carbon-coated 200 mesh grids (Agar Scientific, Stansted, UK). The droplets were allowed to adsorb on the grid for 20 min. Then, the same grids were incubated with 2% uranyl acetate (Polysciences, Warrington, PA, USA) for 5 min and air-dried for obtaining contrasted images of EV. UF-EV were visualized using JEM 1400 TEM (JEOL Ltd. Tokyo, Japan, with Morada TEM CCD camera, Olympus, Hamburg, Germany) at 80 kV. The digital images of EV were captured using a numeric camera (Morada TEM CCD camera, Olympus, Hamburg, Germany).

### Mass-spectrometry and proteomic data analysis

#### Sample preparation and measurement

Trichloroacetic acid (TCA) and sodium deoxycholate (DOC) protocol was used for protein precipitation of the samples. Briefly, 5 µL of 100% TCA-DOC solution was added to 20 µL of EV sample and incubated 20 min at 4 °C. The sample was then centrifuged for 15 min at 17,000 g at RT and the supernatant was discarded. Then 60 µL of 100% acetone was added on the pellet, incubated for 10 min at RT and finally centrifuged for 15 min at 17,000 g at RT. After the supernatant was discarded the washing step was repeated. The precipitate was air-dried on ice under the hood for 10 min. For the analysis 1 µg of protein was injected to an Easy-nLC 1000 system (Thermo Fisher Scientific, Waltham, MA, USA). The sample was eluted from the trap at 250 nL/min to 75 µm ID × 50 cm emitter-column (New Objective, Littleton, MA, USA) packed with C18 material (3 µm, 300 Å particles, Dr Maisch HPLC GmbH, Ammerbuch, Germany). The separating gradient used were 2—35% A 60 min and 40—100% B 5 min (A: 0.1% formic acid (FA), B: 80% ACN + 0.1% FA). Next, the eluted peptides were transferred to a Q Exactive Plus (Thermo Fisher Scientific, Waltham, MA, USA) quadrupole-orbitrap mass spectrometer using nano-electrospray ionization at 2.4 kV, which was applied through liquid-junction. The machine was operated with a top-5 data-dependent acquisition strategy. Briefly, at a resolution setting of R = 70,000 at 200 m/z one 350–1400 m/z MS scan was followed by five higher-energy collisional dissociation fragmentation (normalized collision energy of 26) of 5 most intense ions (z: + 2 to + 6) at R = 17,500. The target values of MS and MS/MS were 3 × 10^6^ and 5 × 10^4^ with 50 ms injection time. The dynamic exclusion was limited to 40 s.

#### Analysis of EV protein enrichment

The MS raw files were processed with the MaxQuant software package using versions 1.6.15.0 and 2.0.3.0. The variable modifications were set for methionine oxidation, asparagine and glutamine deamidation, and protein N-terminal acetylation, while cysteine carbomidomethylation was defined as fixed modification. Label-free quantification (LFQ) was enabled using LFQ and protein minimum ratio count at 1. Search was performed against reference proteomes of *Homo sapiens* and *Bos taurus* using the tryptic digestion rule. Target-decoy approach was used to keep peptide-spectrum match and protein false discovery rate (FDR) below 1%. All other parameters for the analysis were used in their default settings.

The proteins previously identified in exosomes were picked from the dataset for further analysis (ExoCarta 2022). The log transformed LFQ values of protein abundance measured from samples before and after EV isolation were compared. Chosen proteins were visualized in R (v4.1.0) using package ggplot2 (Wickham 2016).

#### UF-EV protein profile analysis of luteal and follicular phase samples

The raw files were processed with MaxQuant software similarly as described above. The EV protein profile acquired from MaxQuant data analysis was further processed in R (v4.1.0) using package named Differential Enrichment Analysis of Proteomics (DEP) (Zhang et al. 2018). Briefly, protein profile data was filtered for proteins that were identified in 2 out of 3 samples in one condition and missing values were imputed using random draws from a manually defined left-shifted Gaussian distribution. Cut-off values used were adjusted *P*-value at 0.05 and log2 fold change at 1.5. Finally, the differences of protein profiles between luteal and follicular phases were determined.

The enriched proteins identified with MS was subjected to Kyoto Encyclopaedia of Genes and Genomes (KEGG) and Gene Ontology (GO) pathway analysis using web-based tool: The Database for Annotation, Visualization, and Integrated Discovery (DAVID) (Huang et al. 2009a, 2009b). Additionally, Gene Set Enrichment Analysis (GSEA) with ClusterProfiler in R (v4.1.0) was performed to visualize specific GO pathways (Wu et al. 2021).

### Statistical analysis

Statistical analysis of NTA measurements were done with Graphpad prism v9.3.0.463 and Microsoft Excel. Data is shown as mean ± standard deviation (SD). The comparisons between the concentrations, size, and ZP of fluorescently labelled and total particles were done using the two-tailed Student t-test. The differences of slaughtered and live cows on different oestrous cycle phases were assessed with two-way ANOVA with post-hoc Tukey multiple comparison test for intergroup comparisons. The differences are statistically significant at *P* ≤ 0.05.

## Results

### Confirmation of EV enrichment strategy

**Fig. 2:**
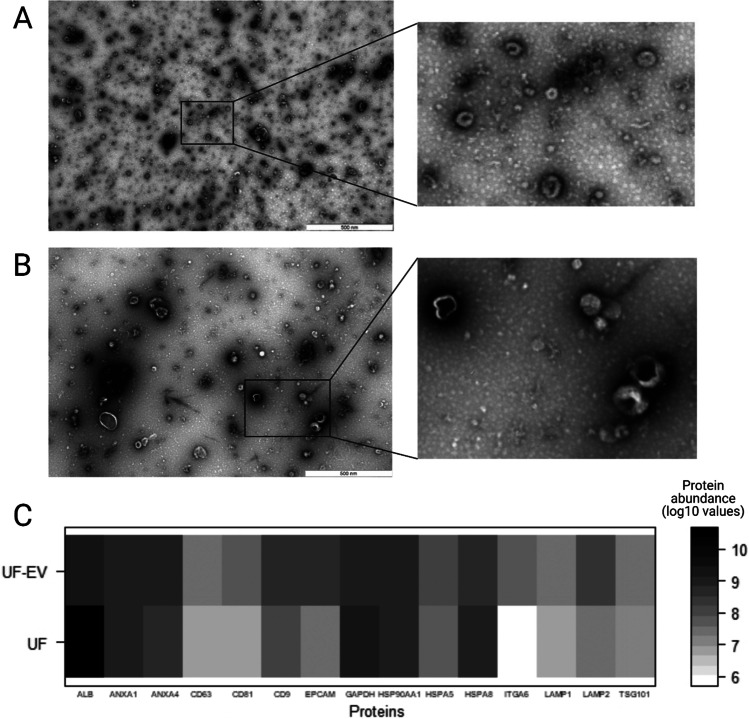
**Validity of bovine uterine fluid (UF) extracellular vesicles (EV) purification.** Transmission electron microscopy (TEM) figure of cow UF-EV can be seen showing cup-shaped vesicular structures in luteal (**A**) and follicular phases (**B**). After isolation of the EV, the UF-EV showed enrichment of proteins Annexin 4 (ANXA4), Cluster determinant (CD)63, CD81, CD9, Epithelial cell adhesion molecule (EPCAM), Heat shock protein family A member (HSPA)5, Integrin subunit alpha 6 (ITGA6), Lysosomal-associated membrane protein (LAMP)1, LAMP2 and Tumour susceptibility gene 101(TSG101), while the protein intensity decreased for Albumin (ALB), Glyceraldehyde-3-phosphate dehydrogenase (GAPDH) and HSPA8 (**C**)

The UF-EV isolation protocol successfully enriched EV fractions in processed samples. UF-EV were detected in the fractions of 6 to 9 after SEC procedures (Supplementary file 3). TEM analysis revealed cup-shaped vesicular structures in UF-EV samples, which is typical morphology for EV (Fig. 2A-B).

MS analysis identified 82 EV related proteins (Supplementary file 4), which have been previously identified in exosomes (ExoCarta 2022) in the UF samples. Forty-four of these proteins were enriched in UF-EV sample. Some examples of enriched EV related proteins in the UF-EV samples were Annexin 4 (ANXA4), Cluster determinant (CD) 63, CD81, CD9, Epithelial cell adhesion molecule (EPCAM), Heat shock protein family A member (HSPA) 5, Integrin subunit alpha 6 (ITGA6), Lysosomal-associated membrane protein (LAMP) 1, LAMP2 and Tumour susceptibility gene 101 (TSG101) (Fig. 2C). However, the enrichment did not happen for 38 proteins previously identified in exosomes, such as Albumin (ALB), Glyceraldehyde-3-phosphate dehydrogenase (GAPDH) and HSPA8 (Fig. 2C).

### EV physical characteristics of UF-EV samples in different phases of the oestrous cycle from live and slaughtered cows

#### Particle concentrations and size profiles of the UF-EV samples at follicular and luteal phases

**Fig. 3:**
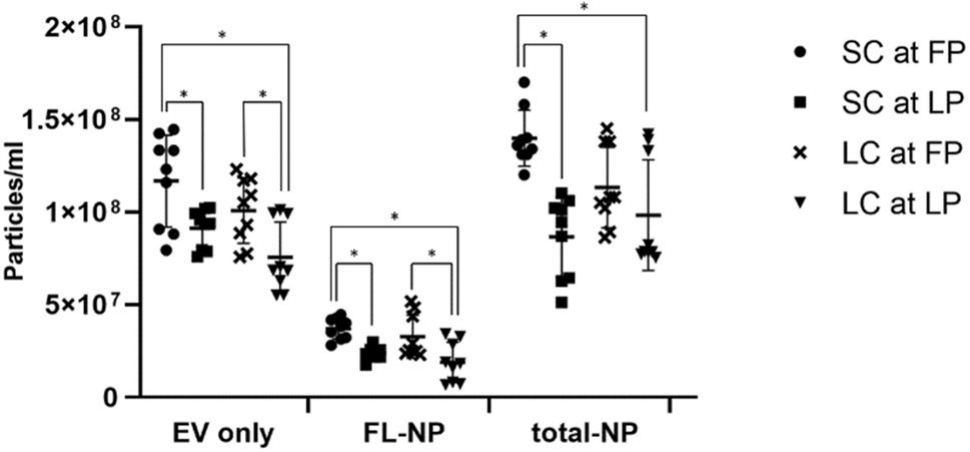
**Particle concentrations (mean ± SD) of the uterine fluid extracellular vesicles (UF-EV).** UF-EV were acquired from slaughtered cows (SC) at follicular phase (FP) (n = 3) and luteal phase (LP) (n = 3) of the oestrous cycle and from live cows (LC) at FP (n = 3) and LP (n = 3) of the oestrous cycle. The EV were isolated with combined tangential flow filtration (TFF) and size exclusion chromatography (SEC) methods. Samples were measured in triplicates. EV samples labelled with CellMask™ Green Plasma Membrane Stain (CMG) were measured in fluorescent mode (FL-NP) at minimum brightness level of 25, while the rest were measured at 30. The intergroup significant differences are marked with asterisk (*) symbol. EV only: EV sample measured in scatter mode; FL-NP: EV samples labelled with CMG dye measured in fluorescent mode; total-NP: EV samples labelled with CMG dye measured in scatter mode

Particle concentrations of the EV samples without and with CMG membrane dye are depicted in Fig. 3. The NTA measurements showed significantly more (*P* ≤ 0.05) particles in slaughtered and live cows at follicular phase compared to luteal phase, except in total-NP (Fig. 3). Also, there were significantly more particles (*P* ≤ 0.05) measured in slaughtered cows at follicular phase compared to live cows at luteal phase (Fig. 3). This result indicates that the amount of EV secreted at the phases of the oestrous cycle differ.

The fluorescently labelled particles showed a lower particle size distribution in fluorescent mode in the range of ~ 75—350 nm than in the scatter mode in all sample types (Supplementary file 5).

The NTA measurements showed significant differences (*P* ≤ 0.05) in mean fluorescent particle sizes of UF-EV between live cows at the follicular phase (220 ± 15 nm) compared to all other sample groups (live cow at luteal phase: 196 ± 15 nm, slaughtered cow at follicular phase: 184 ± 4 nm, slaughtered cow at luteal phase: 188 ± 4 nm). The live cow UF-EV at follicular phase contained larger fluorescently labelled particles compared to its respective luteal phase measurements. Also, the live cow follicular fluorescently labelled UF-EV were larger compared to slaughtered cow UF-EV at different phases of the oestrous cycle.

#### ZP of bovine UF-EV at follicular and luteal phases of the oestrous cycle

**Fig. 4:**
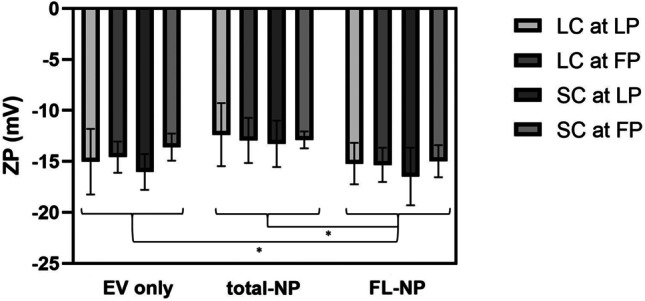
**Zeta potential (ZP) of bovine uterine fluid extracellular vesicles (UF-EV).** UF-EV were acquired from live cows (LC) and slaughtered cows (SC) at follicular (FP) and luteal phases (LP). The EV were isolated with combined tangential flow filtration (TFF) and size exclusion chromatography (SEC) methods. EV samples labelled with CellMask™ Green Plasma Membrane Stain (CMG) in fluorescent mode (FL-NP) were measured at minimum brightness threshold value of 25, while the rest were measured with 30. The significant differences of ZP between measurement groups are marked with asterisk (*) symbol. EV only: EV sample measured in scatter mode, FL-NP: EV samples labelled with CMG dye measured in fluorescent mode, total-NP: EV samples labelled with CMG dye measured in scatter mode

The addition of CMG fluorescent dye to particles led to less negative ZP values for all samples, except for slaughtered cows at follicular phase. The ZP values of fluorescently labelled particles (FL-NP) were significantly more negative (*P* ≤ 0.05) compared to particles measured in scatter modes (Fig. 4). This result indicated that fluorescently labelled particles may have potentially more negative ZP values compared to the rest of the particles in the samples.

### UF-EV protein profile at follicular and luteal phases of the oestrous cycle

**Fig. 5:**
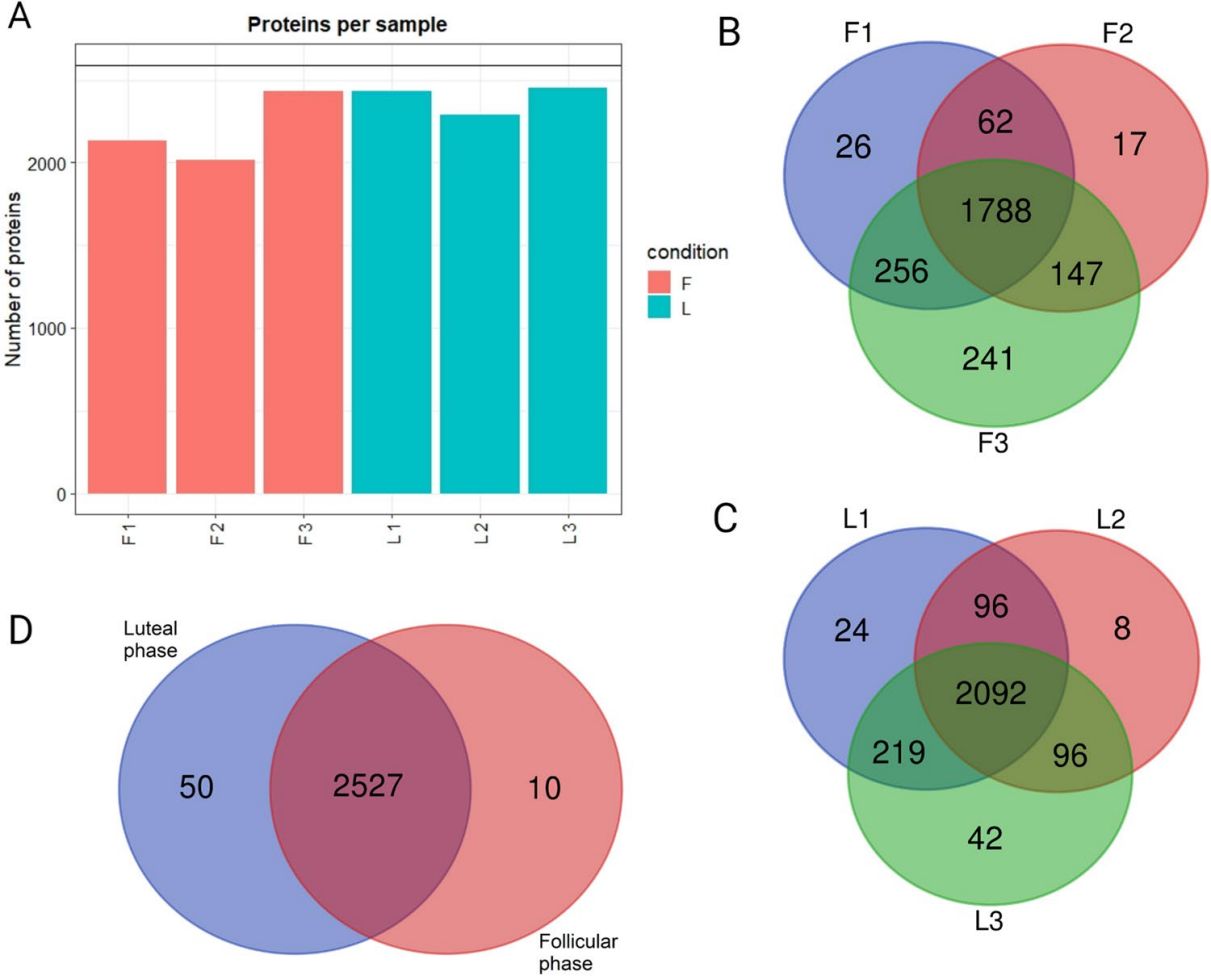
**Identified protein numbers with quantitative mass-spectrometry in live cow samples.** Protein analysis was performed on live cow uterine fluid extracellular vesicle (UF-EV) samples at follicular (F) and luteal phases (L) of the oestrous cycle. Following are the counts per sample: F1 = 2132, F2 = 2014, F3 = 2432, L1 = 2431, L2 = 2292, L3 = 2449 (**A**). An overlap comparison between the follicular phase (**B**) samples (F1, F2, F3) and luteal phase (**C**) samples (L1, L2, L3) showing in total of 1788 and 2092 were common in follicular and luteal phase, respectively. The overlap comparison between follicular and luteal phase showed 2527 identified proteins in common for both (**D**). Venn diagrams were made with webtool: https://bioinformatics.psb.ugent.be/webtools/Venn/

In total, 2587 different proteins were identified in UF-EV samples of the luteal and follicular phases of the oestrous cycle. The total number of proteins identified in different samples differed (Fig. 5A). An overlap comparison shows that 70.5% (n = 1788) and 81.2% (n = 2092) of EV proteins were common in the biological replicates of follicular and luteal phase samples, respectively (Fig. 5B-C). Moreover, 97.7% (n = 2527) of proteins were common for both phases (Fig. 5D). The proteins identified in follicular and luteal phases was subjected to functional analysis with DAVID (Huang et al. 2009a, 2009b). Most commonly, these proteins were found in extracellular exosomes, cytoplasm, plasma membrane, cytosol, and membrane according to GO cellular component terminology. There was in total of 150 KEGG pathways identified in follicular phase and 157 in follicular phase. Most of these pathways (n = 146) were common for both phases and several of them were related to reproductive biology, for example: focal adhesion, gonadotropin releasing hormone signalling, oocyte meiosis, gap junction, vascular endothelial growth factor signalling, adherens junction, regulation of cytoskeleton, phosphoinositide 3 kinase/Akt signalling and chemokine signalling. The only pathways identified related to reproductive biology, which were unique for follicular phase was cyclic adenosine monophosphate signalling pathway and for luteal phase mammalian target of rapamycin pathway.

**Fig. 6:**
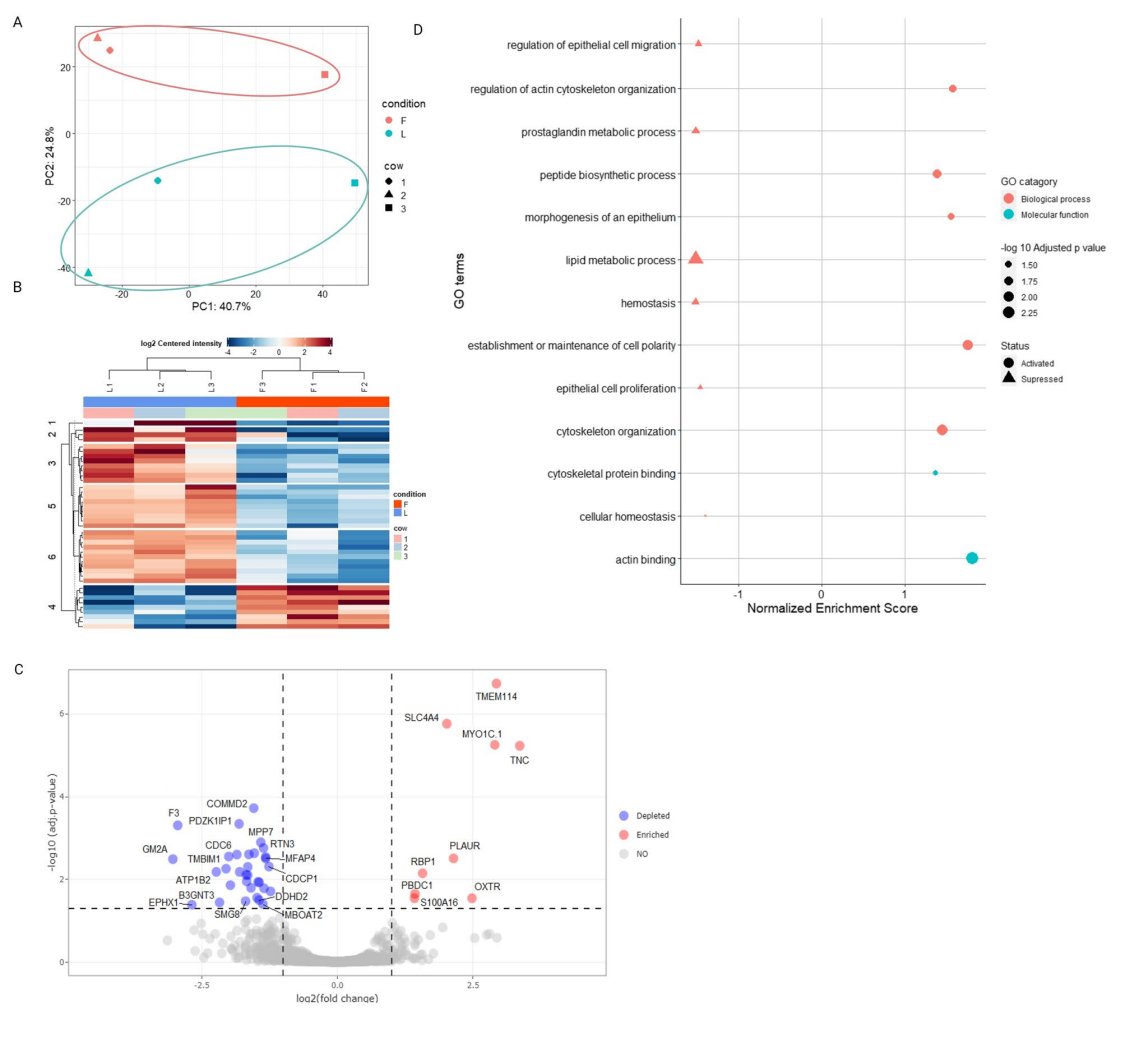
**Differential uterine fluid extracellular vesicles (UF-EV) protein enrichment and functional analysis. **The principal component analysis of 500 most variable proteins in live cows purified UF-EV samples at follicular (F) and luteal (L) phase of the oestrous cycle (**A**). Heatmap of significant proteins (*P* ≤ 0.05) in live cows purified EV samples at F and L phase of the oestrous cycle (**B**). Volcano plot showing differences of the protein enrichment in F and L phases of the oestrous cycle. The proteins that are different between F and L phases are seen in red (enriched in F compared to L) and blue (depleted in F compared to L) with cut-off values of log2 fold change at 1.5 and adjusted *P-*value at 0.05 (**C**). In total of 41 proteins were differentially enriched between the phases (**C**). The GO analysis showed differential enrichment of Gene ontology (GO) biological processes and metabolic function categories, where the activation of GO terms in follicular phase are depicted with the triangle symbol and suppression with a circle (**D**)

The protein abundance of UF-EV at follicular and luteal phases were compared. Our data suggest that there is a clear difference in the protein enrichment between the luteal and follicular phases (Fig. 6A-C). The principal component analysis (PCA) showed a clear separation in the overall protein expression patterns of bovine UF-EV between follicular and luteal phases (Fig. 6A). The heatmap of the significant proteins (*P* ≤ 0.05) showed a clear difference between follicular and luteal phase EV protein expression patterns (Fig. 6B). However, only 41 proteins were differentially enriched (*P* ≤ 0.05) between the two oestrous cycle phases (Fig. 6C; Table 1). In total, 9 proteins were enriched at the follicular phase, while 32 proteins were enriched at the luteal phase (Fig. 6C; Table 1).

**Table 1:** Differentially enriched (*P* ≤ 0.05) proteins between follicular (F) and luteal (L) phases

Protein name	Protein description	F vs L adj. *P*-value*	F-cent PE**	Putative function
ALDH3A2	Aldehyde Dehydrogenase 3 Family Member A2	0.0114	-1.46	Detoxification of aldehydes
ATP1B2	ATPase Na + /K + Transporting Subunit Beta 2	0.00654	-2.23	Ion transport, cell adhesion
B3GNT3	N-acetyllactosaminide beta-1,3-N-acetylglucosaminyltransferase 3	0.0358	-2.17	Metabolic processes
CDC6	Cell Division Cycle 6	0.0025	-1.85	Cell cycle and division, DNA replication, mitosis
CDCP1	CUB Domain Containing Protein 1	0.00486	-1.26	Cell adhesion, cell matrix remodelling
COMMD2	COMM Domain Containing 2	0.000189	-1.54	Cell cycle, apoptosis, oncogenesis, adaptive immunity
CYP51A1	Cytochrome P450 Family 51 Subfamily A Member 1	0.0111	-1.67	Steroid metabolism
DDHD2	DDHD Domain Containing 2	0.0315	-1.44	Lipid metabolism
EFEMP1	EGF Containing Fibulin Extracellular Matrix Protein 1	0.0275	-1.48	Cell adhesion, cell signalling
ENPP6	Ectonucleotide Pyrophosphatase/ Phosphodiesterase 6	0.00549	-2.05	Lipid metabolism
EPHX1	Epoxide Hydrolase 1	0.0407	-2.68	Aromatic hydrocarbons catabolism, detoxification
ERO1A	Endoplasmic Reticulum Oxidoreductase 1 Alpha	0.0137	-1.97	Apoptosis, electron transport
F3	Coagulation Factor III	0.000491	-2.94	Haemostasis
GCNT2	Glucosaminyl (N-Acetyl) Transferase 2	0.00231	-1.53	Metabolic processes
GM2A	GM2 Ganglioside Activator	0.00322	-3.03	Lipid metabolism
HSD17B7	Hydroxysteroid 17-Beta Dehydrogenase 7	0.00247	-1.63	Lipid metabolism, lipid and steroid biosynthesis
ILVBL	IlvB Acetolactate Synthase Like	0.00785	-1.65	Lipid metabolism
INSR	Insulin Receptor	0.0163	-1.35	Carbohydrate metabolism
IRF6	Interferon Regulatory Factor 6	0.0194	-1.23	Epithelial cell proliferation
LAMTOR4	Late Endosomal/Lysosomal Adaptor, MAPK And MTOR Activator 4	0.0159	-1.59	Cell signalling
MBOAT2	Membrane Bound O-Acyltransferase Domain Containing 2	0.0392	-1.37	Lipid biosynthesis and metabolism
MFAP4	Microfibril Associated Protein 4	0.00286	-1.32	Cell adhesion, intercellular interactions
MPP7	MAGUK P55 Scaffold Protein 7	0.00126	-1.41	Cell polarity, tight junction
MSMO1	Methylsterol Monooxygenase 1	0.00769	-1.68	Lipid and steroid metabolism and biosynthesis
MYO1C.1	Myosin IC	0.00000559	2.9	Plasma membrane tension
OXTR	Oxytocin Receptor	0.0283	2.48	Cell signalling
PBDC1	Polysaccharide Biosynthesis Domain Containing 1	0.0222	1.43	*Poorly understood*
PDZK1IP1	PDZK1 Interacting Protein 1	0.000453	-1.81	*Poorly understood*
PLAUR	Urokinase-type plasminogen activator receptor	0.00308	2.14	Cell signalling
RBP1	Retinol Binding Protein 1	0.00709	1.57	Metabolic processes, transport
RTN1	Reticulon 1	0.00311	-1.32	Apoptosis, stress response, ER-Golgi transport
RTN3	Reticulon 3	0.00173	-1.36	Apoptosis, stress response, ER-Golgi transport
S100A16	S100 Calcium Binding Protein A16	0.0283	1.42	Ion transport
SLC4A4	Solute Carrier Family 4 Member 4	0.00000175	2.02	Ion transport
SMG8	SMG8 Nonsense Mediated MRNA Decay Factor	0.0335	-1.69	Nonsense-mediated mRNA decay
TMBIM1	Transmembrane BAX Inhibitor Motif Containing 1	0.00279	-2	Vascular remodelling
TMEM114	Transmembrane Protein 114	0.000000185	2.93	*Poorly understood*
TNC	Tenascin C	0.00000587	3.36	Cell adhesion
TOR2A	Torsin Family 2 Member A	0.0117	-1.44	ATP binding
WFDC2	WAP Four-Disulfide Core Domain 2	0.00654	-1.8	Broad range protease inhibitor
ZDHHC20	Zinc Finger DHHC-Type Palmitoyltransferase 20	0.00499	-1.65	Metabolic processes

^*^ F vs L adj. *P*-value = luteal vs follicular adjusted *P*-value

^**^ F-cent. PE = follicular centered protein expression

Most functional pathways identified are overlapping for both phases, however there are differences in protein abundances between the phases. The identified protein changes in functional pathways of GO and KEGG from follicular and luteal phases was analysed and visualized using R (Wu et al. 2021). The GO enrichment analysis revealed that among the biological processes the supressed of GO categories in the follicular phase compared to the luteal phase were related to cellular metabolic processes (e.g. haemostasis, prostaglandin and lipid metabolic processes), epithelial cell migration and proliferation, while activated in follicular phase in categories related to cytoskeleton organization, morphogenesis of epithelium and maintenance in cellular polarity (Fig. 6D; Supplementary file 6). KEGG pathways showed that EV proteins were involved in different metabolic processes. These pathways were supressed in the follicular phase compared to the luteal phase, such as steroid biosynthesis and fatty acid metabolism and activated in pathways such as nitrogen metabolism (Supplementary file 6).

## Discussion

The dynamic regulation of UF-EV during the oestrous cycle influences the development of endometrial tissue and generates an optimum microenvironment for embryo implantation (O’Neil and Spencer 2021; Simintiras et al. 2019). However, the changes of bovine uterine EV profile during the oestrous cycle remains poorly understood. Despite, many studies using slaughtered cow material for their EV research, there is insufficient information about the changes which occur in EV profiles of different biofluids post-mortem. Therefore, the aim of this study was to characterize UF-EV from live and slaughtered cows, and to evaluate the differences in UF-EV profiles of live and slaughtered cows between follicular and luteal phases of the oestrous cycle using EV concentration and size, and ZP as variables. Finally, we evaluated the difference in protein abundance between the phases of the oestrous cycle in live cows to identify the changes in UF-EV proteome from one phase to another.

In our study, the concentrations of UF-EV between the luteal and follicular phases differed significantly. Moreover, the only UF-EV concentrations that differed between slaughtered and live cows were between slaughtered cows at follicular phase and live cows at luteal phase. So far, no studies have compared slaughtered and live cow UF-EV concentrations and only one study has quantified bovine UF-EV across the oestrous cycle, which could be compared to our results. Hamdi et al. (2021) measured the EV concentrations in bovine UF at different stages of the oestrous cycle. They identified an average of 6.0 × 10^10^ particles/mL regardless of the stage of the oestrous cycle, which is not in agreement with our results where the particle concentrations varied across the oestrous cycle. In human, UF-EV did not differ between proliferative and secretory phases either (Giacomini et al. 2021). However, in line with our findings, sheep UF-EV had significant differences between day 10 and 14 of the oestrous cycle suggesting the involvement of progesterone in controlling UF-EV production (Burns et al. 2018). Multiple factors may attribute to these differences between studies including, but not limited to sample size, the collection method of UF, amount of UF collected, EV isolation/enrichment method and the method used to quantify EV.

UF contains several different molecules such as amino acids, carbohydrates, proteins, and lipids independently from EV (O’Neil and Spencer 2021; Simintiras et al. 2019), which could be potentially co-isolated together with EV when using different EV isolation protocols. However, different methods have been developed to distinguish EV from other types of nanoparticles, one of them being membrane labelling of EV using lipophilic fluorescent dyes. These lipophilic dyes stain the lipid bilayer of the EV, which could be measured with fluorescence NTA (Midekessa et al. 2021). In the current study, we measured the proportion of particles labelled with CMG resulting to lower particle counts compared to measurements without the dye, which indicate that only a proportion of particles measured with the conventional NTA could be EV. This result is in line with previous studies, which have measured proportionately lower fluorescently labelled particles compared to total nanoparticles in cell culture media (Midekessa et al. 2021) and human urine samples (Marcu et al. 2020). Hence, alternative methods to conventional NTA and standardization of these measurements are necessary to accurately measure EV counts needed for biomedical function and for the understanding their biological properties (Hartjes et al. 2019). Moreover, further improvement of EV purification methods would reduce the non-EV particle co-isolation, which would give more accurate results. However, even the alternative methods such as fluorescence NTA has limitations, for example non-EV nanoparticles incorporated with lipids can be labelled with the dye affecting the results.

ZP is the net surface charge of particles in dispersed systems, which can be used to evaluate the activity of EV in biological processes, for example cellular uptake and cytotoxicity (Fröhlich 2012). In biological conditions, EV have a net negative charge due to negatively charged glycosylated proteins in the lipid bilayer (Akagi and Ichiki 2008). However, the exact effects of ZP and their differences in variable physiological conditions is yet to be identified. We measured the ZP of fluorescently labelled and unlabelled particles isolated from bovine UF at the follicular and luteal phases of the oestrous cycle in slaughtered and live cows. The fluorescently labelled particles had more negative ZP values compared to the rest of the particles in our measurements, which is in line with previous results that identified EV charge in biological conditions (Akagi and Ichiki 2008). Moreover, there was no difference in the ZP values of different types of NTA measurements between the two phases of the oestrous cycle or between slaughtered and live cows. Further research is needed to understand the charge or differences of EV surface composition throughout the oestrous cycle or between live and slaughtered cow acquired material, which influence the biological activity of the EV. For example, surface plasmonic analysis (Shpacovitch and Hergenröder 2018) and EV array (Just et al. 2020) could be potential future tools to understand if surface components of EV play a role in physiological processes in the uterus of live or slaughtered cows at different phases of the oestrous cycle.

Our study investigated the differential enrichment of proteins in UF-EV during the follicular and luteal phases of the oestrous cycle in live cows. These EV-related proteins measured can be found inside the EV, incorporated to the EV membrane or in the EV protein corona (Simon et al. 2018; Tóth et al. 2021). Moreover, as mentioned above, only a proportion of particles isolated from the UF could be EV. Some of these non-EV related particles could be protein coagulates, therefore the MS measurements can include some non-EV related proteins.

To the best of our knowledge, this is the only bovine UF-EV proteome analysis to date. Previously, only human UF-EV proteome had been characterized, showing the differences in EV protein enrichment between secretory and proliferative phases of the oestrous cycle (Rai et al. 2021). The study of Rai et al. (2021) investigated the protein composition of UF secretome, soluble secretome, and small EV in fertile and infertile women. Our study investigated only UF-EV proteins in healthy cattle at follicular and luteal phases of the oestrous cycle. Despite the differences in the study designs, overall conclusions of both investigations were similar, i.e., the phase of the cycle influences UF-EV proteome. Our study identified in total of 2587 proteins at luteal and follicular phases of the bovine oestrous cycle, while the study of Rai et al. (2021) identified 240 UF-EV proteins in secretory and proliferative phases. These differences in identifying protein numbers may be related to differences in EV isolation method, MS methodology, and material used. Further, it is important to note that human menstrual cycle differs from bovine oestrous cycle, which can influence the protein abundance at a specific cycle time point and proteomic differences between the two studies. In the study by Rai et al. (2021), the authors identified differentially enriched proteins between secretory and proliferative phases that implicated in antioxidant activity, lipid metabolism, antimicrobial function, mucosal immunity, glycolysis, and coagulation. In our study we identified many of those proteins, which were differentially enriched in human secretory and proliferative phases, however none were altered in bovine UF-EV between follicular and luteal phases of the cycle. For example, in the study of Rai et al. (2021) the authors discovered 32 UF-EV proteins that are more enriched in secretory phase, which are related to regulation of invasion. Out of the 32 proteins, 27 were identified in our study, but none of them were differentially enriched between the follicular and luteal phases of the cycle. Therefore, further studies are needed on bovine and human UF-EV with standardized EV protein analysation methodology to identify protein enrichment between different phases and to compare the results.

Of the 41 differentially expressed proteins in our study, 9 were enriched in follicular phase and 32 in luteal phase (Table 1). Compared to follicular phase, the bovine UF-EV proteome at the luteal phase was mainly enriched with proteins responsible for endometrial proliferation and differentiation. Moreover, some of the differentially enriched proteins were reported in different KEGG pathways, one of them being steroid biosynthesis pathway.

The enriched proteins on steroid biosynthesis pathway were Cytochrome P450 family 51 subfamily A member 1 (CYP51A1), Methylsterol monooxygenase 1 (MSMO1, also known as SC4MOL) and Hydroxysteroid 17-beta dehydrogenase 7 (HSD17B7), which were significantly enriched during the luteal phase. These enzymes in the steroid biosynthesis pathway are responsible for conversion of lanosterol to cholesterol (Supplementary file 7), which is essential for several biological functions such as steroidogenesis and maintaining cell homeostasis (Chatuphonprasert et al. 2018). Cholesterol is found in cell membranes, where it increases the cell stiffness, impermeability to water and ions, and coupled with proteins are important in cellular signalling. Furthermore, cholesterol is essential for synthesis of all steroid hormones, which all regulate homeostasis of cells in reproductive tract (Cortes et al. 2014).

New studies support an idea that progesterone acts via the endometrium by influencing EV production and its cargo in uterine lumen (Burns et al. 2018), which subsequently regulates conceptus growth and elongation in pregnancy (Burns et al. 2018; Liu et al. 2020; Vilella et al. 2015). Therefore, cholesterol synthesis might be essential for preparing uterine environment for embryo growth. In our study we observed enrichment of proteins responsible for cholesterol production in luteal phase, which have been shown to be crucial for the development of endometrium (Méndez-Tepepa et al. 2020; Shehu et al. 2008; Tavares Pereira et al. 2022). For example, CYP51A1 has been identified in endometrium and myometrium, where it has a role in the uterine decidualization process and eventually embryo implantation (Méndez-Tepepa et al. 2020). Therefore, the increased enrichment of CYP51A1 at luteal phase UF-EV may facilitate cell-to-cell communication of endometrial cells during peri-implantation period to induce endometrial growth.

Recently, studies have described that UF-EV carrying bioactive substances are not only important for endometrial, but also for early embryonic development (Tannetta et al. 2014; Bridi et al 2020). Several studies have shown that embryos are able to uptake maternal EV, while influencing embryos (Vilella et al. 2015; Burns et al. 2016). Moreover, when the embryo reaches to uterus at day 4 to 5 after ovulation in the beginning of luteal phase of the oestrous cycle (Forde et al. 2011; Sponchiado et al. 2017), it has been shown to start partaking in endometrium-embryo crosstalk (Burns et al. 2014; Dissanayake et al. 2021b; Es-Haghi et al. 2019). Thus, UF-EV have an important role in embryo-maternal interface. Previous studies have identified the proteins CYP51A1 and MSMO1 on steroid biosynthesis pathway in cumulus cells but not in oocytes (Su et al. 2009), which suggests that oocytes are unable to synthesize cholesterol. As cholesterol receptors have not been identified in oocytes (Sato et al. 2003; Seli et al. 2014), the oocyte requires a cooperation with cumulus cells for their cholesterol need. By the blastocyst stage embryos acquire the ability to synthesise their own cholesterol (Sato et al. 2003), however with increased cell proliferation and membrane formation rates the embryonic and foetal cells have elevated cholesterol requirements (Cortes et al. 2014). The lack of cholesterol influences embryo quality and can lead to embryonic diseases (Cortes et al. 2014). Therefore, the enzymes to synthesise cholesterol, which can be taken up via UF-EV by embryo, are essential for embryo development from the blastocyst stage being responsible for promoting embryo growth and quality.

Moreover, cholesterol is a precursor for steroid hormone production such as progesterone and oestradiol, which is essential for establishing pregnancy (Cortes et al. 2014). HSD17B7 has previously been found in CL of many species, including ruminants (Shehu et al. 2008). The HSD17B7 abundance is known to increase after the early luteal stage contributing to CL maturation and production of sex hormones (Tavares Pereira et al. 2022). Due to the ability of EV to function as autocrine and paracrine messengers’, UF-EV would support both growth and development of endometrium and embryos, when present *in vitro*. Therefore, it is likely that these enriched proteins have a functional significance in UF-EV during the luteal phase and requires further research to understand the mechanisms that prepares the uterine environment for conceptus growth during the pre-implantation period.

## Conclusions

Our study confirmed the presence of EV in bovine UF, where the EV concentration and protein profile varied between follicular and luteal phases of the oestrous cycle. Protein profile analysis of EV revealed differences in protein profiles at follicular and luteal phases, suggesting that EV may modulate the uterine microenvironment across the oestrous cycle towards helping with remodelling and establishing cell polarity in endometrium during different stages of the reproductive cycle. Taken together, our research provides the basis for understanding the changes in the bovine UF-EV profile during the oestrous cycle. Further research could be conducted to determine the necessary changes in uterine EV for successful conception and the possibility of using this information to improve fertility in cattle.

## Supplementary Information

Below is the link to the electronic supplementary material.

Supplementary file1 (DOCX 18 KB)

Supplementary file2 (DOCX 1327 KB)

Supplementary file3 (DOCX 50 KB)

Supplementary file4 (DOCX 23 KB)

Supplementary file5 (DOCX 215 KB)

Supplementary file6 (DOCX 35 KB)

Supplementary file7 (DOCX 164 KB)

## Data Availability

The data that support the findings of this study is available from the corresponding author upon reasonable request. The mass spectrometry proteomics data have been deposited to the ProteomeXchange Consortium via the PRIDE (Perez-Riverol et al. 2022) partner repository with the dataset identifier PXD038002.

## Abbreviations

*ALB*: Albumin
*ANXA4*: Annexin 4
*BSA*: Bovine serum albumin
*CD*: Cluster determinant
*CL*: Corpus luteum
*CMG*: CellMask™ Green Plasma Membrane Stain
*CYP51A1*: Cytochrome P450 family 51 subfamily A member 1
*DAVID*: Database for Annotation, Visualization, and Integrated Discovery
*DEP*: Differential Enrichment Analysis of Proteomics
*DOC*: Sodium deoxycholate
*EEC*: Endometrial epithelial cells
*EPCAM*: Epithelial cell adhesion molecule
*EV*: Extracellular vesicles
*F*: Follicular
*FDR*: False discovery rate
*FL-NP*: Fluorescently labelled particles
*FL-NTA*: Fluorescence nanoparticle tracking analysis
*fps*: Frames per second
*GAPDH*: Glyceraldehyde-3-phosphate dehydrogenase
*GO*: Gene Ontology
*GSEA*: Gene Set Enrichment Analysis
*HSD17B7*: Hydroxysteroid 17-beta dehydrogenase 7
*HSPA*: Heat shock protein family A member
*ISEV*: International Society for Extracellular Vesicles
*ITGA6*: Integrin subunit alpha 6
*KEGG*: Kyoto Encyclopaedia of Genes and Genomes
*L*: Luteal
*LAMP*: Lysosomal-associated membrane protein
*LC*: Live cows
*LFQ*: Label-free quantification
*MS*: Mass-spectrometry
*MSMO1*: Methylsterol monooxygenase 1
*NTA*: Nanoparticle tracking analysis
*PBS*: Phosphate buffered saline
*PCA*: Principal component analysis
*RT*: Room temperature
*SC*: Slaughtered cows
*SD*: Standard deviation
*SEC*: Size exclusion chromatography
*TCA*: Trichloroacetic acid
*TEM*: Transmission electron microscopy
*TFF*: Tangential flow filtration
*TSG101*: Tumor susceptibility gene 101
*UF*: Uterine fluid
*ZP*: Zeta potential

## References

[CR1] Akagi T, Ichiki T (2008). Cell electrophoresis on a chip: what can we know from the changes in electrophoretic mobility?. Anal Bioanal Chem.

[CR2] Andrade GM, Bridi A, Gimenes LU (2019). Extracellular vesicles and its advances infemale reproduction. Anim Reprod.

[CR3] Arosh JA, Parent J, Chapdelaine P (2002). Expression of cyclooxygenases 1 and 2 and prostaglandin E synthase in bovine endometrial tissue during the estrous cycle. Bio Reprod.

[CR4] Bridi A, Perecin F, Silveira JCD (2020). Extracellular vesicles mediated early embryo-maternal interactions. Int J Mol Sci.

[CR5] Burns G, Brooks K, Wildung M (2014). Extracellular vesicles in luminal fluid of the ovine uterus. PLoS ONE.

[CR6] Burns GW, Brooks KE, Spencer TE (2016). Extracellular vesicles originate from the conceptus and uterus during early pregnancy in sheep. Biol Reprod.

[CR7] Burns GW, Brooks KE, O'Neil EV (2018). Progesterone effects on extracellular vesicles in the sheep uterus. Biol Reprod.

[CR8] Caballero J, Frenette G, Sullivan R (2010). Post testicular sperm maturational changes in the bull: important role of the epididymosomes and prostasomes. Vet Med Int.

[CR9] Chae JI, Kim J, Lee SG et al (2011) Proteomic analysis of pregnancy-related proteins from pig uterus endometrium during pregnancy. Proteome Sci 9(41). 10.1186/1477-5956-9-4 10.1186/1477-5956-9-41 PMC3162492 21791079

[CR10] Chatuphonprasert W, Jarukamjorn K, Ellinger I (2018). Physiology and pathophysiology of steroid biosynthesis, transport and metabolism in the human placenta. Front Pharmacol.

[CR11] Cortes VA, Busso D, Maiz A (2014). Physiological and pathological implications of cholesterol. Front Biosci (landmark Ed).

[CR12] DesCôteaux L, Gnemmi G, Colloton J (2009) Ultrasonography of the bovine female genital tract. Vet Clin North Am Food Anim Pract 25(3):733–52, Table of Contents. 10.1016/j.cvfa.2009.07.009 10.1016/j.cvfa.2009.07.009 19825441

[CR13] Dissanayake K, Midekessa G, Lättekivi F, Fazeli A (2021). Measurement of the Size and concentration and zeta potential of extracellular vesicles using nanoparticle tracking analyzer. Methods Mol Biol.

[CR14] Dissanayake K, Nõmm M, Lättekivi F (2021). Oviduct as a sensor of embryo quality: deciphering the extracellular vesicle (EV)-mediated embryo-maternal dialogue. J Mol Med (berl).

[CR15] Es-Haghi M, Godakumara K, Häling A (2019). Specific trophoblast transcripts transferred by extracellular vesicles affect gene expression in endometrial epithelial cells and may have a role in embryo-maternal crosstalk. Cell Commun Signal.

[CR16] ExoCarta: List of top 100 proteins that are often identified in exosomes. http://exocarta.org/exosome_markers_new. Accessed 3 Feb 2022

[CR17] Faulkner S, Elia G, O’Boyle P (2013). Composition of the bovine uterine proteome is associated with stage of cycle and concentration of systemic progesterone. Proteomics.

[CR18] Forde N, Beltman ME, Lonergan P (2011). Oestrous cycles in Bos taurus cattle. Anim Reprod Sci.

[CR19] Fröhlich E (2012). The role of surface charge in cellular uptake and cytotoxicity of medical nanoparticles. Int J Nanomed.

[CR20] Giacomini E, Scotti GM, Vanni VS (2021). Global transcriptomic changes occur in uterine fluid-derived extracellular vesicles during the endometrial window for embryo implantation. Hum Reprod.

[CR21] Hamdi M, Cañon-Beltrán K, Mazzarella R (2021). Characterization and profiling analysis of bovine oviduct and uterine extracellular vesicles and their miRNA cargo through the estrous cycle. FASEB J.

[CR22] Hartjes TA, Mytnyk S, Jenster GW (2019). Extracellular vesicle quantification and characterization: common methods and emerging approaches. Bioengineering (Basel).

[CR23] Hasan MM, Reshi QUA, Lättekivi F (2021). Bovine follicular fluid derived extracellular vesicles modulate the viability, capacitation and acrosome reaction of bull spermatozoa. Biology (Basel).

[CR24] Herrero C, de la Fuente A, Casas-Arozamena C (2019). Extracellular vesicles-based biomarkers represent a promising liquid biopsy in endometrial cancer. Cancers (Basel).

[CR25] Huang DW, Sherman BT, Lempicki RA (2009). Bioinformatics enrichment tools: paths toward the comprehensive functional analysis of large gene lists. Nucleic Acids Res.

[CR26] Huang DW, Sherman BT, Lempicki RA (2009). Systematic and integrative analysis of large gene lists using DAVID Bioinformatics Resources. Nature Protoc.

[CR27] Just J, Yan Y, Farup J (2020). Blood flow-restricted resistance exercise alters the surface profile, miRNA cargo and functional impact of circulating extracellular vesicles. Sci Rep.

[CR28] Kusama K, Nakamura K, Bai R (2018). Intrauterine exosomes are required for bovine conceptus implantation. Biochem Biophys Res Commun.

[CR29] Lange-Consiglio A, Lazzari B, Pizzi F (2020). Amniotic microvesicles impact hatching and pregnancy percentages of in vitro bovine embryos and blastocyst microRNA expression versus in vivo controls. Sci Rep.

[CR30] Liu C, Yao W, Yao J, Li L (2020). Endometrial extracellular vesicles from women with recurrent implantation failure attenuate the growth and invasion of embryos. Fertil Steril.

[CR31] Marcu IC, Eberhard N, Yerly A (2020). Isolation of human small extracellular vesicles and tracking of their uptake by retinal pigment epithelial cells in vitro. Int J Mol Sci.

[CR32] Martins T, Pugliesi G, Sponchiado M (2018). Perturbations in the uterine luminal fluid composition are detrimental to pregnancy establishment in cattle. J Anim Sci Biotechnol.

[CR33] Méndez-Tepepa M, Zepeda-Pérez D, Nicolás-Toledo L (2020). Inferring lanosterol functions in the female rabbit reproductive tract based on the immunolocalization of lanosterol 14-demethylase and farnesoid beta-receptor. Acta Histochem.

[CR34] Midekessa G, Godakumara K, Dissanayake K (2021). Characterization of extracellular vesicles labelled with a lipophilic dye using fluorescence nanoparticle tracking analysis. Membranes (Basel).

[CR35] Nakamura K, Kusama K, Ideta A (2019). Effects of miR-98 in intrauterine extracellular vesicles on maternal immune regulation during the peri-implantation period in cattle. Sci Rep.

[CR36] O’Neil EV, Spencer TE (2021). Insights into the lipidome and primary metabolome of the uterus from day 14 cyclic and pregnant sheep. Bio Reprod.

[CR37] Perez-Riverol Y, Bai J, Bandla C, Hewapathirana S (2022). The PRIDE database resources in 2022: A Hub for mass spectrometry-based proteomics evidences. Nucleic Acids Res.

[CR38] Pioltine EM, Machado MF, da Silveira JC (2020). Can extracellular vesicles from bovine ovarian follicular fluid modulate the in-vitro oocyte meiosis progression similarly to the CNP-NPR2 system?. Theriogenology.

[CR39] Rai A, Poh QH, Fatmous M (2021). Proteomic profiling of human uterine extracellular vesicles reveal dynamic regulation of key players of embryo implantation and fertility during menstrual cycle. Proteomics.

[CR40] Reese ST, Franco GA, Poole RK (2020). Pregnancy loss in beef cattle: A meta-analysis. Anim Reprod Sci.

[CR41] Reshi QUA, Hasan MM, Dissanayake K, Fazeli A (2021). Isolation of extracellular vesicles (EVs) using benchtop size exclusion chromatography (SEC) columns. Methods Mol Biol.

[CR42] Ruiz-González I, Xu J, Wang X (2015). Exosomes, endogenous retroviruses and toll-like receptors: pregnancy recognition in ewes. Reproduction.

[CR43] Sato N, Kawamura K, Fukuda J (2003). Expression of LDL receptor and uptake of LDL in mouse preimplantation embryos. Mol Cell Endocrinol.

[CR44] Schobers G, Koeck R, Pellaers D (2021). Liquid biopsy: state of reproductive medicine and beyond. Hum Reprod.

[CR45] Seli E, Babayev E, Collins SC (2014). Minireview: Metabolism of female reproduction: regulatory mechanisms and clinical implications. Mol Endocrinol.

[CR46] Shehu A, Mao J, Gibori GB (2008). Prolactin receptor-associated protein/17beta-hydroxysteroid dehydrogenase type 7 gene (Hsd17b7) plays a crucial role in embryonic development and fetal survival. Mol Endocrinol.

[CR47] Shpacovitch V, Hergenröder R (2018). Optical and surface plasmonic approaches to characterize extracellular vesicles. A Review Anal Chim Acta.

[CR48] Simintiras CA, Forde N (2017). Understanding the uterine environment in early pregnancy in cattle: How have the omics enhanced our knowledge?. Anim Reprod.

[CR49] Simintiras CA, Sánchez JM, McDonald M, Lonergan P (2019). The biochemistry surrounding bovine conceptus elongation†. Biol Reprod.

[CR50] Simon C, Greening DW, Bolumar D (2018). Extracellular vesicles in human reproduction in health and disease. Endocr Rev.

[CR51] Spencer TE (2014). Biological roles of uterine glands in pregnancy. Semin Reprod Med.

[CR52] Sponchiado M, Gomes NS, Fontes PK (2017). Pre-hatching embryo-dependent and -independent programming of endometrial function in cattle. PLoS ONE.

[CR53] Su YQ, Sugiura K, Eppig JJ (2009). Mouse oocyte control of granulosa cell development and function: paracrine regulation of cumulus cell metabolism. Semin Reprod Med.

[CR54] Sullivan R, Saez F, Girouard J, Frenette G (2005). Role of exosomes in sperm maturation during the transit along the male reproductive tract. Blood Cells Mol Dis.

[CR55] Tannetta D, Dragovic R, Alyahyaei Z, Southcombe J (2014). Extracellular vesicles and reproduction-promotion of successful pregnancy. Cell Mol Immunol.

[CR56] Tavares Pereira M, Papa P, Reichler IM (2022). Luteal expression of factors involved in the metabolism and sensitivity to oestrogens in the dog during pregnancy and in non-pregnant cycle. Reprod Domest Anim.

[CR57] Théry C, Witwer KW, Aikawa E (2018). Minimal information for studies of extracellular vesicles 2018 (MISEV2018): a position statement of the International Society for Extracellular Vesicles and update of the MISEV2014 guidelines. J Extracell Vesicles.

[CR58] Tóth EÁ, Turiák L, Visnovitz T (2021). Formation of a protein corona on the surface of extracellular vesicles in blood plasma. J Extracell Vesicles.

[CR59] Valdmann M, Kurykin J, Kaart T (2018). Relationships between plasma insulin-like growth factor-1 and insulin concentrations in multiparous dairy cows with cytological endometritis. Vet Rec.

[CR60] Vilella F, Moreno-Moya JM, Balaguer N (2015). Hsa-miR-30d, secreted by the human endometrium, is taken up by the pre-implantation embryo and might modify its transcriptome. Development.

[CR61] Waldmann A, Raud A (2016). Comparison of a lateral flow milk progesterone test with enzyme immunoassay as an aid for reproductive status determination in cows. Vet Rec.

[CR62] Wickham H (2016) ggplot2: Elegant Graphics for Data Analysis. Springer-Verlag New York. ISBN 978–3–319–24277–4. https://ggplot2.tidyverse.org

[CR63] Wu T, Hu E, Xu M et al (2021) clusterProfiler, 4.0: A universal enrichment tool for interpreting omics data. Innovation (N Y) 2(3):100141. 10.1016/j.xinn.2021.100141 10.1016/j.xinn.2021.100141 PMC8454663 34557778

[CR64] Zhang X, Smits A, van Tilburg G (2018). Proteome-wide identification of ubiquitin interactions using UbIA-MS. Nat Protoc.

