# Supplementary material for: Characterization of bovine uterine fluid extracellular vesicles proteomic profiles at follicular and luteal phases of the oestrous cycle
Source: Vet Res Commun. 2022 Dec 22;47(2):885–900. doi: 10.1007/s11259-022-10052-3 (PMC10209254; doi:10.1007/s11259-022-10052-3)
Supplement: Supplementary file 1 — Supplementary file1 (DOCX 18 KB) [file 11259_2022_10052_MOESM1_ESM.docx]

**Supplementary file 1: Evaluation of ovarian structures in slaughtered and live cows to determine the phase of the oestrous cycle.** The phase of the oestrous cycle was determined in live cows using ultrasonographic (US) assessment of ovarian structures and P4 rapid milk progesterone test. The phase of the oestrous cycle was determined in slaughtered cows by morphological evaluation of the ovarian structures.

| **Status of the cow** | **US measurements of ovarian structures** | **Morphological assessment of ovarian structures *** | **Results of P4 rapid milk progesterone test (progesterone level)**** | **Phase of the oestrous cycle** |
| --- | --- | --- | --- | --- |
| Live cow 1 | Left ovary: F 2 cm, several F <0.5 cm  Right ovary: several F <0.5 cm | - | Low | Follicular phase |
| Live cow 2 | Left ovary: F 1.8 cm  Right ovary: F 1.6 cm | - | Low | Follicular phase |
| Live cow 3 | Left ovary: F 1.9 cm, F 1.0 cm  Right ovary: several F <0.5 cm | - | Low | Follicular phase |
| Live cow 4 | Left ovary: CL 1.9x1.55 cm; several F <0.5 cm; 5 F 0.8-1.0 cm Right ovary: CL 2.3 cm; F 1.97 cm | - | High | Luteal phase |
| Live cow 5 | Left ovary: F 1.95x1.53 cm; several F <0.5 cm Right ovary: CL 2.73x1.75 cm | - | High | Luteal phase |
| Live cow 6 | Left ovary: several F <0.5 cm  Right ovary: CL 2.86 cm with a hole of 1.14 cm | - | High | Luteal phase |
| Slaughtered cow 1 | - | Left ovary: several F <0.05 cm  Right ovary: several F <0.5 cm; CL 1.0 cm with external colour white, protuberance <0.1 cm, covered by connective tissue, internal colour yellow with clear margins between CL and ovarian stroma, vascularization not visible | - | Follicular phase |
| Slaughtered cow 2 | - | Left ovary: F 2.0 cm; CL 1.5 cm with a hole of 0.8 cm, external colour yellow, protuberance 0.1 cm, covered by connective tissue, internal colour yellow with clear margins between CL and ovarian stroma, vascularisation not visible  Right ovary: several F <0.5 cm | - | Follicular phase |
| Slaughtered cow 3 | - | Right ovary: several F <0.5 cm  Left ovary: F 1.3 cm; CL 0.7 cm with external colour yellow, protuberance 0.1 cm, internal colour light yellow with clear margins between CL and ovarian stroma, covered by connective tissue, regressing blood vessels at periphery apex and base; CL 0.4 cm with external colour bloody, no protuberance, recently ovulated and not covered by epithelial cells, vascularisation not visible, internal colour bloody, no clear margins between CL and ovarian stroma | - | Follicular phase |
| Slaughtered cow 4 | - | Left ovary: F 2.4 cm; CL 2.3 cm with external colour tan, protuberance 0.5 cm, covered by luteal tissue, internal colour tan-orange, well developed blood vessels at periphery and apex, distinct margins between CL and ovarian stroma  Right ovary: several F <0.5 cm | - | Luteal phase |
| Slaughtered cow 5 | - | Left ovary: CL 1.2 cm with external colour brownish red, vascularisation not visible, protuberance 0.2 cm, covered by luteal tissue, internal colour at apex reddish brown and the remaining CL reddish orange, distinct margins between CL and ovarian stroma  Right ovary: F 1.8 cm; several F <0.5 cm | - | Luteal phase |
| Slaughtered cow 6 | - | Left ovary: F 3.0 cm  Right ovary: CL 2.8 cm with external colour tan, protuberance 0.4 cm, covered by luteal tissue, growing vessels appear only at periphery, internal colour tan, distinct margins between CL and ovarian stroma | - | Luteal phase |

* The morphological assessment of ovarian structures was based on the protocol by Arosh et al. (2002).
** The P4 rapid test results were interpretedaccording to Waldmann and Raud (2016): low progesterone = LFT test line darker than the reference line and high progesterone = LFT test line lighter than the reference line, including an invisible test line.
US = ultrasonography, F = follicle(s), CL = corpus luteum
