# Supplementary material for: Characterization of bovine uterine fluid extracellular vesicles proteomic profiles at follicular and luteal phases of the oestrous cycle
Source: Vet Res Commun. 2022 Dec 22;47(2):885–900. doi: 10.1007/s11259-022-10052-3 (PMC10209254; doi:10.1007/s11259-022-10052-3)
Supplement: Supplementary file 2 — Supplementary file2 (DOCX 1327 KB) [file 11259_2022_10052_MOESM2_ESM.docx]

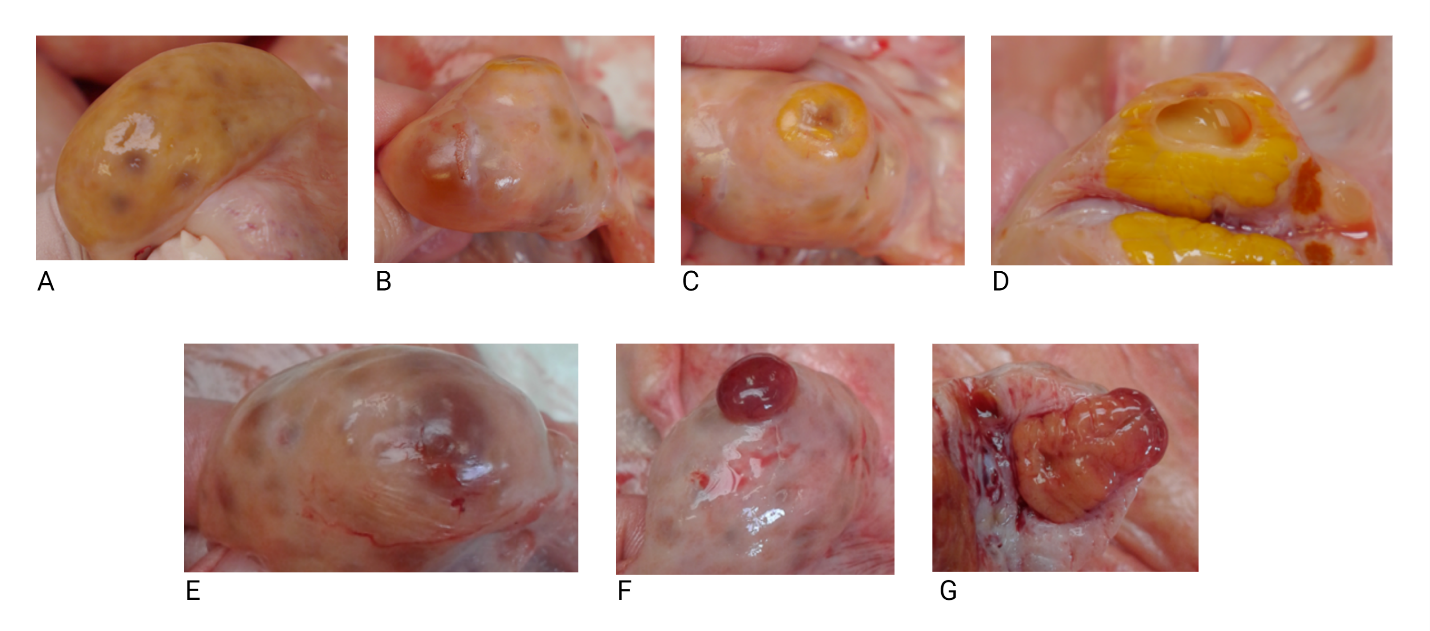


**Supplementary file 2: Examples of ovarian structures evaluation.** The cow uterus was determined to be sampled in follicular phase in the first row (A-D). The right ovary only contained several <0.5 cm follicles (A). The left ovary contained one 2.0 cm follicle and one yellow *corpus luteum* (CL) (B). The CL protuberance was measured to be 0.1 cm and covered by connective tissue (C). After dissection, the 1.5 cm yellow CL showed clear margins between CL and ovarian stroma without visible vascularisation (D). The cow uterus was determined to be sampled in luteal phase in the second row (E-G). The right ovary contained a 1.8 cm follicle and several <0.5 cm follicles (E). The left ovary contained a brownish red coloured CL with a protuberance of 0.2 cm (F). After dissection, the 1.2 cm CL apex was coloured reddish brown and the remaining CL reddish orange with distinct margins between CL and ovarian stroma (G).
