# Supplementary material for: Characterization of bovine uterine fluid extracellular vesicles proteomic profiles at follicular and luteal phases of the oestrous cycle
Source: Vet Res Commun. 2022 Dec 22;47(2):885–900. doi: 10.1007/s11259-022-10052-3 (PMC10209254; doi:10.1007/s11259-022-10052-3)
Supplement: Supplementary file 3 — Supplementary file3 (DOCX 50 KB) [file 11259_2022_10052_MOESM3_ESM.docx]

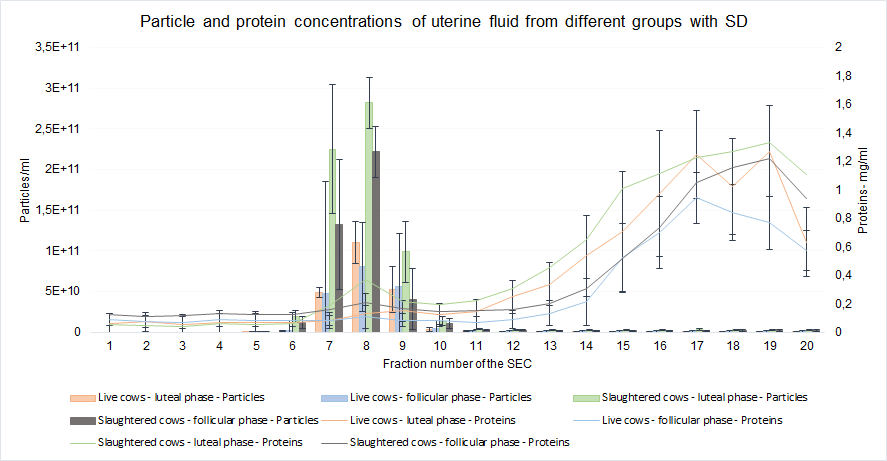


**Supplementary file 3: Particle and protein concentrations of uterine fluid (UF) from different groups.** Particle and protein concentrations with standard deviation (mean ± SD) were measured as described above at point 2.4. There is seen a clear separation of proteins and particles after extracellular vesicles (EV) isolation using combined tangential flow filtration and size exclusion chromatography (SEC) methods. UF-EV were detected in the fractions of 6 to 9.
