# Supplementary material for: Characterization of bovine uterine fluid extracellular vesicles proteomic profiles at follicular and luteal phases of the oestrous cycle
Source: Vet Res Commun. 2022 Dec 22;47(2):885–900. doi: 10.1007/s11259-022-10052-3 (PMC10209254; doi:10.1007/s11259-022-10052-3)
Supplement: Supplementary file 4 — Supplementary file4 (DOCX 23 KB) [file 11259_2022_10052_MOESM4_ESM.docx]

**Supplementary file 4: Identified extracellular vesicles (EV) related protein enrichment or depletion after EV isolation from uterine fluid (UF).** UF sample protein abundance was measured before and after EV isolation using mass-spectrometry. Thereafter, the comparison was made between the measurements showing depleted or enriched protein abundance after EV isolation.

| **Protein description** | **Protein name** | **Protein abundance before EV isolation*** | **Protein abundance after EV isolation*** | **Status** |
| --- | --- | --- | --- | --- |
| Alpha-2-macroglobulin | A2M | 8.40 | 7.57 | Depleted |
| ATP-citrate synthase | ACLY | 7.29 | 7.25 | Depleted |
| Actin. cytoplasmic 1 | ACTB | 8.48 | 8.35 | Depleted |
| Actin. cytoplasmic 2 | ACTG1 | 9.61 | 9.52 | Depleted |
| Alpha-actinin-4 | ACTN4 | 6.65 | 5.95 | Depleted |
| Adenosylhomocysteinase | AHCY | 8.48 | 7.64 | Depleted |
| Serum albumin | ALB | 10.36 | 9.16 | Depleted |
| Fructose-bisphosphate aldolase | ALDOA | 8.92 | 8.23 | Depleted |
| Annexin A1 | ANXA1 | 8.92 | 8.87 | Depleted |
| Annexin A11 | ANXA11 | 7.50 | 8.04 | Enriched |
| Annexin A2 | ANXA2 | 9.46 | 10.05 | Enriched |
| Annexin A4 | ANXA4 | 8.68 | 9.11 | Enriched |
| ADP-ribosylation factor 1 | ARF1 | 8.31 | 8.40 | Enriched |
| Sodium/potassium-transporting ATPase subunit alpha-1 | ATP1A1 | 7.24 | 8.25 | Enriched |
| T-complex protein 1 subunit beta | CCT2 | 7.98 | 8.03 | Enriched |
| Chaperonin containing TCP1 subunit 5 | CCT5 | 7.96 | 8.05 | Enriched |
| CD63 antigen | CD63 | 6.88 | 7.54 | Enriched |
| CD81 antigen | CD81 | 6.81 | 7.61 | Enriched |
| CD9 antigen | CD9 | 7.99 | 8.76 | Enriched |
| Cell division control protein 42 homolog | CDC42 | 8.33 | 8.41 | Enriched |
| Clathrin heavy chain 1 | CLTC | 8.03 | 8.82 | Enriched |
| Elongation factor 1-alpha 1 | EEF1A1 | 9.19 | 9.14 | Depleted |
| Elongation factor 2 | EEF2 | 8.71 | 8.44 | Depleted |
| EH domain containing 4 | EHD4 | 7.20 | 7.45 | Enriched |
| Epithelial cell adhesion molecule | EPCAM | 7.51 | 8.54 | Enriched |
| Ezrin | EZR | 8.83 | 8.68 | Depleted |
| Fatty acid synthase | FASN | 8.00 | 8.07 | Enriched |
| Filamin A | FLNA | 7.90 | 7.86 | Depleted |
| Flotillin-1 | FLOT1 | 6.26 | 7.07 | Enriched |
| Fibronectin | FN1 | 7.80 | 7.38 | Depleted |
| Glyceraldehyde-3-phosphate dehydrogenase | GAPDH | 9.16 | 8.84 | Depleted |
| Rab GDP dissociation inhibitor beta | GDI2 | 8.61 | 8.19 | Depleted |
| Isoform Gnas-2 of Guanine nucleotide-binding protein G(s) subunit alpha isoforms short | GNAS | 7.29 | 7.70 | Enriched |
| Guanine nucleotide-binding protein G(I)/G(S)/G(T) subunit beta-1 | GNB1 | 8.12 | 8.70 | Enriched |
| Guanine nucleotide-binding protein G(I)/G(S)/G(T) subunit beta-2 | GNB2 | 7.61 | 8.31 | Enriched |
| Gelsolin | GSN | 8.75 | 8.30 | Depleted |
| Heat shock protein HSP 90-alpha | HSP90AA1 | 9.09 | 9.00 | Depleted |
| Heat shock protein HSP 90-beta | HSP90AB1 | 8.84 | 8.87 | Enriched |
| Endoplasmic reticulum chaperone BiP | HSPA5 | 7.71 | 8.12 | Enriched |
| Heat shock cognate 71 kDa protein | HSPA8 | 8.85 | 8.64 | Depleted |
| Integrin subunit alpha 6 | ITGA6 | 6.02 | 7.70 | Enriched |
| Integrin beta-1 | ITGB1 | 7.05 | 7.95 | Enriched |
| Karyopherin subunit beta 1 | KPNB1 | 8.34 | 8.10 | Depleted |
| Lysosome-associated membrane glycoprotein 1 | LAMP1 | 6.68 | 7.50 | Enriched |
| Lysosomal associated membrane protein 2 | LAMP2 | 7.32 | 8.21 | Enriched |
| L-lactate dehydrogenase A chain | LDHA | 8.87 | 8.08 | Depleted |
| L-lactate dehydrogenase | LDHB | 8.82 | 8.40 | Depleted |
| Galectin-3-binding protein | LGALS3BP | 8.09 | 8.52 | Enriched |
| Lactadherin | MFGE8 | 7.06 | 7.58 | Enriched |
| Moesin | MSN | 8.08 | 7.44 | Depleted |
| Programmed cell death 6 interacting protein | PDCD6IP | 7.71 | 7.86 | Enriched |
| Profilin-1 | PFN1 | 9.02 | 8.28 | Depleted |
| Phosphoglycerate kinase 1 | PGK1 | 8.64 | 7.94 | Depleted |
| Peptidyl-prolyl cis-trans isomerase A | PPIA | 9.14 | 8.55 | Depleted |
| Peroxiredoxin-1 | PRDX1 | 9.16 | 8.97 | Depleted |
| Peroxiredoxin-2 | PRDX2 | 8.76 | 8.50 | Depleted |
| Prostaglandin F2 receptor inhibitor | PTGFRN | 6.19 | 7.10 | Enriched |
| RAB14 protein | RAB14 | 7.46 | 7.59 | Enriched |
| Ras-related protein Rab-5A | RAB5A | 6.85 | 7.01 | Enriched |
| RAB5B. member RAS oncogene family | RAB5B | 7.86 | 7.90 | Enriched |
| Ras-related protein Rab-5C | RAB5C | 7.13 | 7.31 | Enriched |
| Ras-related protein Rab-7a | RAB7A | 7.51 | 7.76 | Enriched |
| Ras-related protein Rab-8A | RAB8A | 7.69 | 7.83 | Enriched |
| Ras-related C3 botulinum toxin substrate 1 | RAC1 | 8.33 | 8.03 | Depleted |
| GTP-binding nuclear protein Ran | RAN | 8.58 | 8.52 | Depleted |
| Ras-related protein Rap-1b | RAP1B | 6.84 | 7.28 | Enriched |
| Transforming protein RhoA | RHOA | 8.39 | 8.49 | Enriched |
| Syndecan binding protein | SDCBP | 7.44 | 8.08 | Enriched |
| STOM protein | STOM | 7.50 | 7.96 | Enriched |
| T-complex protein 1 subunit alpha | TCP1 | 7.93 | 8.00 | Enriched |
| Thrombospondin-1 | THBS1 | 6.72 | 6.11 | Depleted |
| Triosephosphate isomerase | TPI1 | 8.90 | 7.85 | Depleted |
| TSG101 protein | TSG101 | 7.12 | 7.49 | Enriched |
| Tubulin alpha chain | TUBA1A | 8.45 | 8.92 | Enriched |
| Tubulin alpha-1B chain | TUBA1B | 9.16 | 9.45 | Enriched |
| Tubulin alpha-1C chain | TUBA1C | 7.56 | 7.91 | Enriched |
| Ubiquitin-like modifier-activating enzyme 1 | UBA1 | 8.10 | 7.71 | Depleted |
| Isoform Short of 14-3-3 protein beta/alpha | YWHAB | 8.79 | 8.49 | Depleted |
| 14-3-3 protein epsilon | YWHAE | 9.18 | 8.88 | Depleted |
| 14-3-3 protein eta | YWHAH | 7.96 | 7.79 | Depleted |
| 14-3-3 protein theta | YWHAQ | 8.71 | 8.20 | Depleted |
| 14-3-3 protein zeta/delta | YWHAZ | 9.11 | 8.63 | Depleted |

* Log10 transformed values
