# Supplementary material for: Characterization of bovine uterine fluid extracellular vesicles proteomic profiles at follicular and luteal phases of the oestrous cycle
Source: Vet Res Commun. 2022 Dec 22;47(2):885–900. doi: 10.1007/s11259-022-10052-3 (PMC10209254; doi:10.1007/s11259-022-10052-3)
Supplement: Supplementary file 5 — Supplementary file5 (DOCX 215 KB) [file 11259_2022_10052_MOESM5_ESM.docx]

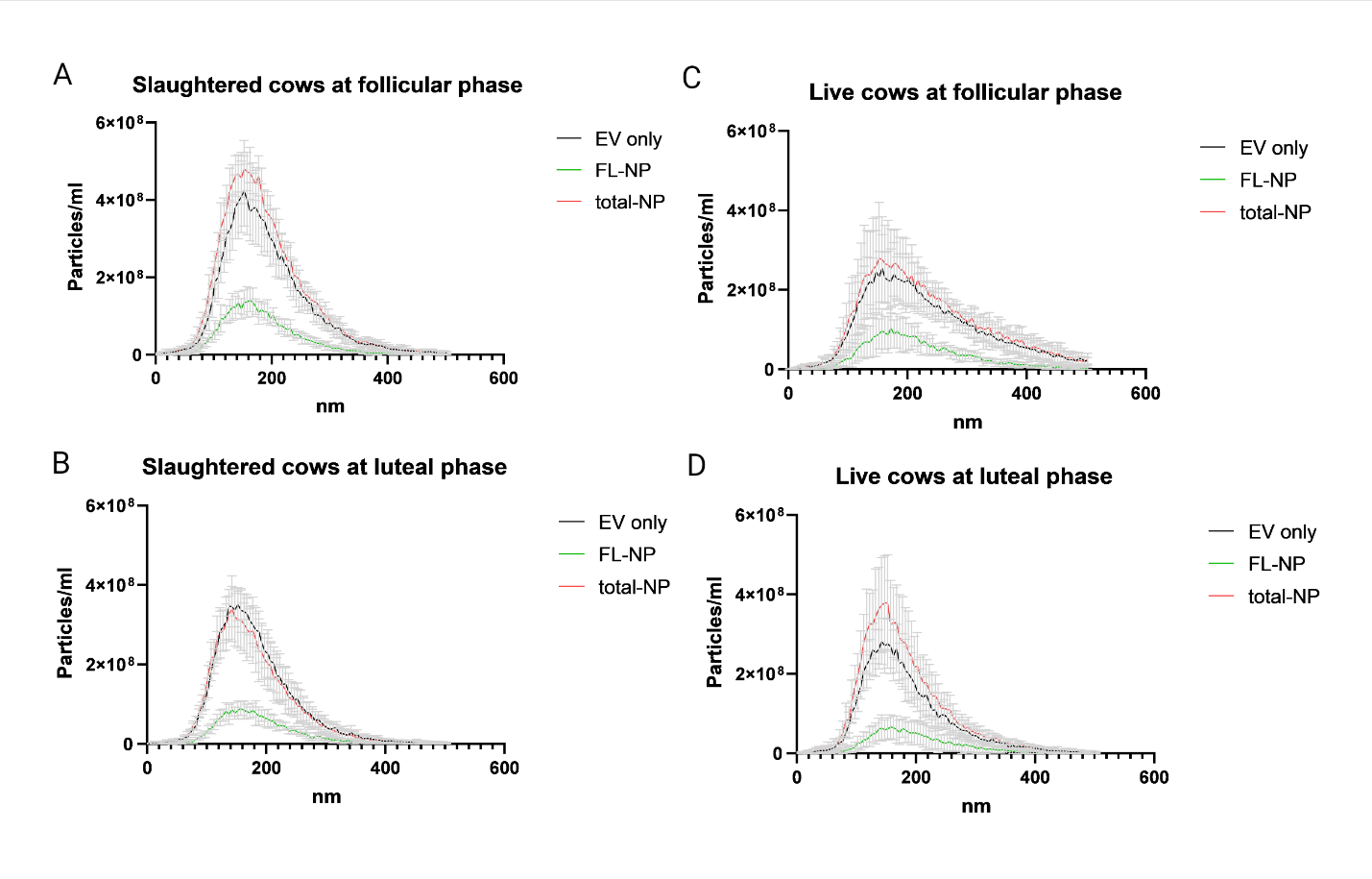


**Supplementary file 5: Size distribution of uterine fluid extracellular vesicles (UF-EV).** UF-EV were acquired from slaughtered (A, B) and live cows (C, D) at follicular (A, C) and luteal phases (B; D) of the oestrous cycle. EV were isolated with combined tangential flow filtration (TFF) and size exclusion chromatography (SEC) methods. EV samples labelled with CellMask™ Green Plasma Membrane Stain (CMG) were measured in fluorescent mode (FL-NP) at the minimum brightness level of 25, while the rest were measured at 30. Each condition had three biological replicates and measurements are presented as mean ± standard deviation (SD). EV only: EV sample measured in scatter mode, FL-NP: EV samples labelled with CMG dye measured in fluorescent mode, total-NP: EV samples labelled with CMG dye measured in scatter mode.
