# Supplementary material for: Characterization of bovine uterine fluid extracellular vesicles proteomic profiles at follicular and luteal phases of the oestrous cycle
Source: Vet Res Commun. 2022 Dec 22;47(2):885–900. doi: 10.1007/s11259-022-10052-3 (PMC10209254; doi:10.1007/s11259-022-10052-3)
Supplement: Supplementary file 6 — Supplementary file6 (DOCX 35 KB) [file 11259_2022_10052_MOESM6_ESM.docx]

**Supplementary file 6: Total list of identified significantly differentially (*P* ≤ 0.05) activated or suppressed Gene Ontology (GO) cellular function and biological processes or Kyoto Encyclopaedia of Genes and Genomes (KEGG) pathways in follicular phase compared to luteal phase.** Normalized enrichment score shows the activation or suppression of a pathway in follicular phase compared to luteal phase.

| **Pathway ID** | **Description** | **Normalized enrichment score** | ***P*-adjusted value** | **Category** | **Status** |
| --- | --- | --- | --- | --- | --- |
| bta00062 | Fatty acid elongation | -1.51 | 0.027 | KEGG | Supressed |
| bta00100 | Steroid biosynthesis | -1.59 | 0.014 | KEGG | Supressed |
| bta00140 | Steroid hormone biosynthesis | -1.62 | 0.008 | KEGG | Supressed |
| bta00220 | Arginine biosynthesis | 1.60 | 0.030 | KEGG | Activated |
| bta00510 | N-Glycan biosynthesis | -1.55 | 0.025 | KEGG | Supressed |
| bta00564 | Glycerophospholipid metabolism | -1.57 | 0.021 | KEGG | Supressed |
| bta00565 | Ether lipid metabolism | -1.53 | 0.026 | KEGG | Supressed |
| bta00910 | Nitrogen metabolism | 1.64 | 0.018 | KEGG | Activated |
| bta00920 | Sulphur metabolism | -1.45 | 0.035 | KEGG | Supressed |
| bta00970 | Aminoacyl-tRNA biosynthesis | 1.83 | 0.005 | KEGG | Activated |
| bta00980 | Metabolism of xenobiotics by cytochrome P450 | -1.51 | 0.033 | KEGG | Supressed |
| bta01100 | Metabolic pathways | -1.24 | 0.034 | KEGG | Supressed |
| bta01212 | Fatty acid metabolism | -1.59 | 0.016 | KEGG | Supressed |
| bta03010 | Ribosome | 2.33 | 0.001 | KEGG | Activated |
| bta03040 | Spliceosome | -1.40 | 0.039 | KEGG | Supressed |
| bta03050 | Proteasome | 1.99 | 0.002 | KEGG | Activated |
| bta04060 | Cytokine-cytokine receptor interaction | -1.48 | 0.038 | KEGG | Supressed |
| bta04614 | Renin-angiotensin system | -1.55 | 0.020 | KEGG | Supressed |
| bta04620 | Toll-like receptor signaling pathway | 1.65 | 0.024 | KEGG | Activated |
| bta04714 | Thermogenesis | -1.49 | 0.029 | KEGG | Supressed |
| bta04960 | Aldosterone-regulated sodium reabsorption | -1.67 | 0.007 | KEGG | Supressed |
| bta05171 | Coronavirus disease - COVID-19 | 1.48 | 0.006 | KEGG | Activated |
| bta05204 | Chemical carcinogenesis - DNA adducts | -1.67 | 0.007 | KEGG | Supressed |
| bta05221 | Acute myeloid leukaemia | 1.54 | 0.047 | KEGG | Activated |
| bta05310 | Asthma | 1.63 | 0.021 | KEGG | Activated |
| GO:0001516 | prostaglandin biosynthetic process | -1.56 | 0.015 | Biological process | Supressed |
| GO:0001836 | release of cytochrome c from mitochondria | 1.80 | 0.009 | Biological process | Activated |
| GO:0001837 | epithelial to mesenchymal transition | -1.58 | 0.013 | Biological process | Supressed |
| GO:0002009 | morphogenesis of an epithelium | 1.56 | 0.032 | Biological process | Activated |
| GO:0002526 | acute inflammatory response | -1.65 | 0.006 | Biological process | Supressed |
| GO:0002639 | positive regulation of immunoglobulin production | -1.41 | 0.049 | Biological process | Supressed |
| GO:0003007 | heart morphogenesis | -1.46 | 0.050 | Biological process | Supressed |
| GO:0003341 | cilium movement | -1.48 | 0.020 | Biological process | Supressed |
| GO:0003735 | structural constituent of ribosome | 1.58 | 0.034 | Cellular function | Activated |
| GO:0003755 | peptidyl-prolyl cis-trans isomerase activity | -1.56 | 0.020 | Cellular function | Supressed |
| GO:0003779 | actin binding | 1.81 | 0.004 | Cellular function | Activated |
| GO:0006412 | translation | 1.45 | 0.009 | Biological process | Activated |
| GO:0006629 | lipid metabolic process | -1.51 | 0.005 | Biological process | Supressed |
| GO:0006643 | membrane lipid metabolic process | -1.60 | 0.014 | Biological process | Supressed |
| GO:0006665 | sphingolipid metabolic process | -1.54 | 0.025 | Biological process | Supressed |
| GO:0006691 | leukotriene metabolic process | -1.55 | 0.007 | Biological process | Supressed |
| GO:0006692 | prostanoid metabolic process | -1.51 | 0.032 | Biological process | Supressed |
| GO:0006693 | prostaglandin metabolic process | -1.51 | 0.032 | Biological process | Supressed |
| GO:0006873 | cellular ion homeostasis | -1.45 | 0.037 | Biological process | Supressed |
| GO:0007009 | plasma membrane organization | -1.48 | 0.041 | Biological process | Supressed |
| GO:0007010 | cytoskeleton organization | 1.46 | 0.008 | Biological process | Activated |
| GO:0007015 | actin filament organization | 1.58 | 0.021 | Biological process | Activated |
| GO:0007163 | establishment or maintenance of cell polarity | 1.76 | 0.011 | Biological process | Activated |
| GO:0007183 | SMAD protein complex assembly | -1.47 | 0.016 | Biological process | Supressed |
| GO:0007596 | blood coagulation | -1.52 | 0.033 | Biological process | Supressed |
| GO:0007599 | haemostasis | -1.52 | 0.033 | Biological process | Supressed |
| GO:0007610 | behaviour | 1.69 | 0.016 | Biological process | Activated |
| GO:0007626 | locomotory behaviour | 1.69 | 0.028 | Biological process | Activated |
| GO:0008092 | cytoskeletal protein binding | 1.37 | 0.041 | Cellular function | Activated |
| GO:0008154 | actin polymerization or depolymerization | 1.64 | 0.023 | Biological process | Activated |
| GO:0008637 | apoptotic mitochondrial changes | 1.74 | 0.014 | Biological process | Activated |
| GO:0009612 | response to mechanical stimulus | 1.66 | 0.025 | Biological process | Activated |
| GO:0010232 | vascular transport | -1.42 | 0.043 | Biological process | Supressed |
| GO:0010447 | response to acidic pH | -1.49 | 0.011 | Biological process | Supressed |
| GO:0010594 | regulation of endothelial cell migration | -1.53 | 0.026 | Biological process | Supressed |
| GO:0010595 | positive regulation of endothelial cell migration | -1.49 | 0.031 | Biological process | Supressed |
| GO:0010632 | regulation of epithelial cell migration | -1.48 | 0.036 | Biological process | Supressed |
| GO:0010638 | positive regulation of organelle organization | 1.84 | 0.003 | Biological process | Activated |
| GO:0010639 | negative regulation of organelle organization | 1.56 | 0.037 | Biological process | Activated |
| GO:0010640 | regulation of platelet-derived growth factor receptor signalling pathway | -1.42 | 0.042 | Biological process | Supressed |
| GO:0010719 | negative regulation of epithelial to mesenchymal transition | -1.42 | 0.033 | Biological process | Supressed |
| GO:0016830 | carbon-carbon lyase activity | 1.59 | 0.043 | Cellular function | Activated |
| GO:0016859 | cis-trans isomerase activity | -1.56 | 0.020 | Cellular function | Supressed |
| GO:0016879 | ligase activity. forming carbon-nitrogen bonds | 1.63 | 0.021 | Cellular function | Activated |
| GO:0016918 | retinal binding | 1.54 | 0.029 | Cellular function | Activated |
| GO:0019725 | cellular homeostasis | -1.40 | 0.050 | Biological process | Supressed |
| GO:0019841 | retinol binding | 1.54 | 0.029 | Cellular function | Activated |
| GO:0030010 | establishment of cell polarity | 1.65 | 0.028 | Biological process | Activated |
| GO:0030029 | actin filament-based process | 1.63 | 0.005 | Biological process | Activated |
| GO:0030036 | actin cytoskeleton organization | 1.77 | 0.003 | Biological process | Activated |
| GO:0030165 | PDZ domain binding | 1.56 | 0.046 | Cellular function | Activated |
| GO:0030258 | lipid modification | -1.46 | 0.046 | Biological process | Supressed |
| GO:0030509 | BMP signalling pathway | -1.43 | 0.029 | Biological process | Supressed |
| GO:0030838 | positive regulation of actin filament polymerization | 1.66 | 0.029 | Biological process | Activated |
| GO:0030851 | granulocyte differentiation | -1.43 | 0.031 | Biological process | Supressed |
| GO:0030879 | mammary gland development | -1.44 | 0.048 | Biological process | Supressed |
| GO:0031110 | regulation of microtubule polymerization or depolymerization | 1.62 | 0.037 | Biological process | Activated |
| GO:0031420 | alkali metal ion binding | 1.53 | 0.049 | Cellular function | Activated |
| GO:0032271 | regulation of protein polymerization | 1.66 | 0.015 | Biological process | Activated |
| GO:0032273 | positive regulation of protein polymerization | 1.73 | 0.013 | Biological process | Activated |
| GO:0032956 | regulation of actin cytoskeleton organization | 1.58 | 0.029 | Biological process | Activated |
| GO:0033043 | regulation of organelle organization | 1.80 | 0.001 | Biological process | Activated |
| GO:0034205 | amyloid-beta formation | -1.49 | 0.019 | Biological process | Supressed |
| GO:0034383 | low-density lipoprotein particle clearance | 1.49 | 0.050 | Biological process | Activated |
| GO:0035088 | establishment or maintenance of apical/basal cell polarity | 1.68 | 0.032 | Biological process | Activated |
| GO:0040014 | regulation of multicellular organism growth | -1.53 | 0.017 | Biological process | Supressed |
| GO:0043043 | peptide biosynthetic process | 1.39 | 0.018 | Biological process | Activated |
| GO:0043491 | protein kinase B signalling | -1.46 | 0.047 | Biological process | Supressed |
| GO:0043534 | blood vessel endothelial cell migration | -1.58 | 0.016 | Biological process | Supressed |
| GO:0043535 | regulation of blood vessel endothelial cell migration | -1.61 | 0.011 | Biological process | Supressed |
| GO:0043589 | skin morphogenesis | 1.56 | 0.020 | Biological process | Activated |
| GO:0043604 | amide biosynthetic process | 1.38 | 0.016 | Biological process | Activated |
| GO:0045010 | actin nucleation | 1.77 | 0.015 | Biological process | Activated |
| GO:0045732 | positive regulation of protein catabolic process | 1.58 | 0.034 | Biological process | Activated |
| GO:0045766 | positive regulation of angiogenesis | -1.44 | 0.048 | Biological process | Supressed |
| GO:0045777 | positive regulation of blood pressure | 1.59 | 0.012 | Biological process | Activated |
| GO:0046456 | icosanoid biosynthetic process | -1.68 | 0.004 | Biological process | Supressed |
| GO:0046457 | prostanoid biosynthetic process | -1.56 | 0.015 | Biological process | Supressed |
| GO:0046835 | carbohydrate phosphorylation | -1.47 | 0.015 | Biological process | Supressed |
| GO:0048008 | platelet-derived growth factor receptor signalling pathway | -1.42 | 0.042 | Biological process | Supressed |
| GO:0048407 | platelet-derived growth factor binding | 1.56 | 0.021 | Cellular function | Activated |
| GO:0048762 | mesenchymal cell differentiation | -1.50 | 0.034 | Biological process | Supressed |
| GO:0050000 | chromosome localization | 1.63 | 0.027 | Biological process | Activated |
| GO:0050435 | amyloid-beta metabolic process | -1.49 | 0.019 | Biological process | Supressed |
| GO:0050673 | epithelial cell proliferation | -1.46 | 0.045 | Biological process | Supressed |
| GO:0050817 | coagulation | -1.53 | 0.026 | Biological process | Supressed |
| GO:0051017 | actin filament bundle assembly | 1.58 | 0.030 | Biological process | Activated |
| GO:0051087 | chaperone binding | 1.58 | 0.031 | Cellular function | Activated |
| GO:0051258 | protein polymerization | 1.62 | 0.020 | Biological process | Activated |
| GO:0051303 | establishment of chromosome localization | 1.63 | 0.027 | Biological process | Activated |
| GO:0051310 | metaphase plate congression | 1.63 | 0.027 | Biological process | Activated |
| GO:0051493 | regulation of cytoskeleton organization | 1.83 | 0.002 | Biological process | Activated |
| GO:0051494 | negative regulation of cytoskeleton organization | 1.73 | 0.015 | Biological process | Activated |
| GO:0051495 | positive regulation of cytoskeleton organization | 1.67 | 0.013 | Biological process | Activated |
| GO:0051607 | defence response to virus | 1.57 | 0.038 | Biological process | Activated |
| GO:0051897 | positive regulation of protein kinase B signalling | -1.49 | 0.036 | Biological process | Supressed |
| GO:0055082 | cellular chemical homeostasis | -1.51 | 0.018 | Biological process | Supressed |
| GO:0060485 | mesenchyme development | -1.61 | 0.012 | Biological process | Supressed |
| GO:0061099 | negative regulation of protein tyrosine kinase activity | 1.64 | 0.024 | Biological process | Activated |
| GO:0061180 | mammary gland epithelium development | -1.52 | 0.020 | Biological process | Supressed |
| GO:0061245 | establishment or maintenance of bipolar cell polarity | 1.68 | 0.032 | Biological process | Activated |
| GO:0061572 | actin filament bundle organization | 1.58 | 0.030 | Biological process | Activated |
| GO:0070507 | regulation of microtubule cytoskeleton organization | 1.56 | 0.040 | Biological process | Activated |
| GO:0071467 | cellular response to pH | -1.50 | 0.015 | Biological process | Supressed |
| GO:0071468 | cellular response to acidic pH | -1.49 | 0.011 | Biological process | Supressed |
| GO:0071772 | response to BMP | -1.43 | 0.029 | Biological process | Supressed |
| GO:0071773 | cellular response to BMP stimulus | -1.43 | 0.029 | Biological process | Supressed |
| GO:0072132 | mesenchyme morphogenesis | -1.46 | 0.018 | Biological process | Supressed |
| GO:0090199 | regulation of release of cytochrome c from mitochondria | 1.72 | 0.014 | Biological process | Activated |
| GO:0090200 | positive regulation of release of cytochrome c from mitochondria | 1.56 | 0.022 | Biological process | Activated |
| GO:0097435 | supramolecular fibre organization | 1.47 | 0.018 | Biological process | Activated |
| GO:0110053 | regulation of actin filament organization | 1.61 | 0.022 | Biological process | Activated |
| GO:0140546 | defence response to symbiont | 1.57 | 0.038 | Biological process | Activated |
| GO:0150104 | transport across blood-brain barrier | -1.42 | 0.043 | Biological process | Supressed |
| GO:1901655 | cellular response to ketone | 1.63 | 0.039 | Biological process | Activated |
| GO:1902003 | regulation of amyloid-beta formation | -1.41 | 0.041 | Biological process | Supressed |
| GO:1902903 | regulation of supramolecular fibre organization | 1.86 | 0.004 | Biological process | Activated |
| GO:1902904 | negative regulation of supramolecular fibre organization | 1.74 | 0.014 | Biological process | Activated |
| GO:1902905 | positive regulation of supramolecular fibre organization | 1.65 | 0.016 | Biological process | Activated |
| GO:1902991 | regulation of amyloid precursor protein catabolic process | -1.47 | 0.023 | Biological process | Supressed |
| GO:1903364 | positive regulation of cellular protein catabolic process | 1.56 | 0.037 | Biological process | Activated |
| GO:1904018 | positive regulation of vasculature development | -1.44 | 0.048 | Biological process | Supressed |
| GO:1905477 | positive regulation of protein localization to membrane | -1.59 | 0.014 | Biological process | Supressed |
| GO:1990000 | amyloid fibril formation | 1.49 | 0.049 | Biological process | Activated |
| GO:2001234 | negative regulation of apoptotic signalling pathway | 1.80 | 0.010 | Biological process | Activated |
| GO:2001243 | negative regulation of intrinsic apoptotic signalling pathway | 1.65 | 0.035 | Biological process | Activated |
