# Supplementary material for: Characterization of bovine uterine fluid extracellular vesicles proteomic profiles at follicular and luteal phases of the oestrous cycle
Source: Vet Res Commun. 2022 Dec 22;47(2):885–900. doi: 10.1007/s11259-022-10052-3 (PMC10209254; doi:10.1007/s11259-022-10052-3)
Supplement: Supplementary file 7 — Supplementary file7 (DOCX 164 KB) [file 11259_2022_10052_MOESM7_ESM.docx]

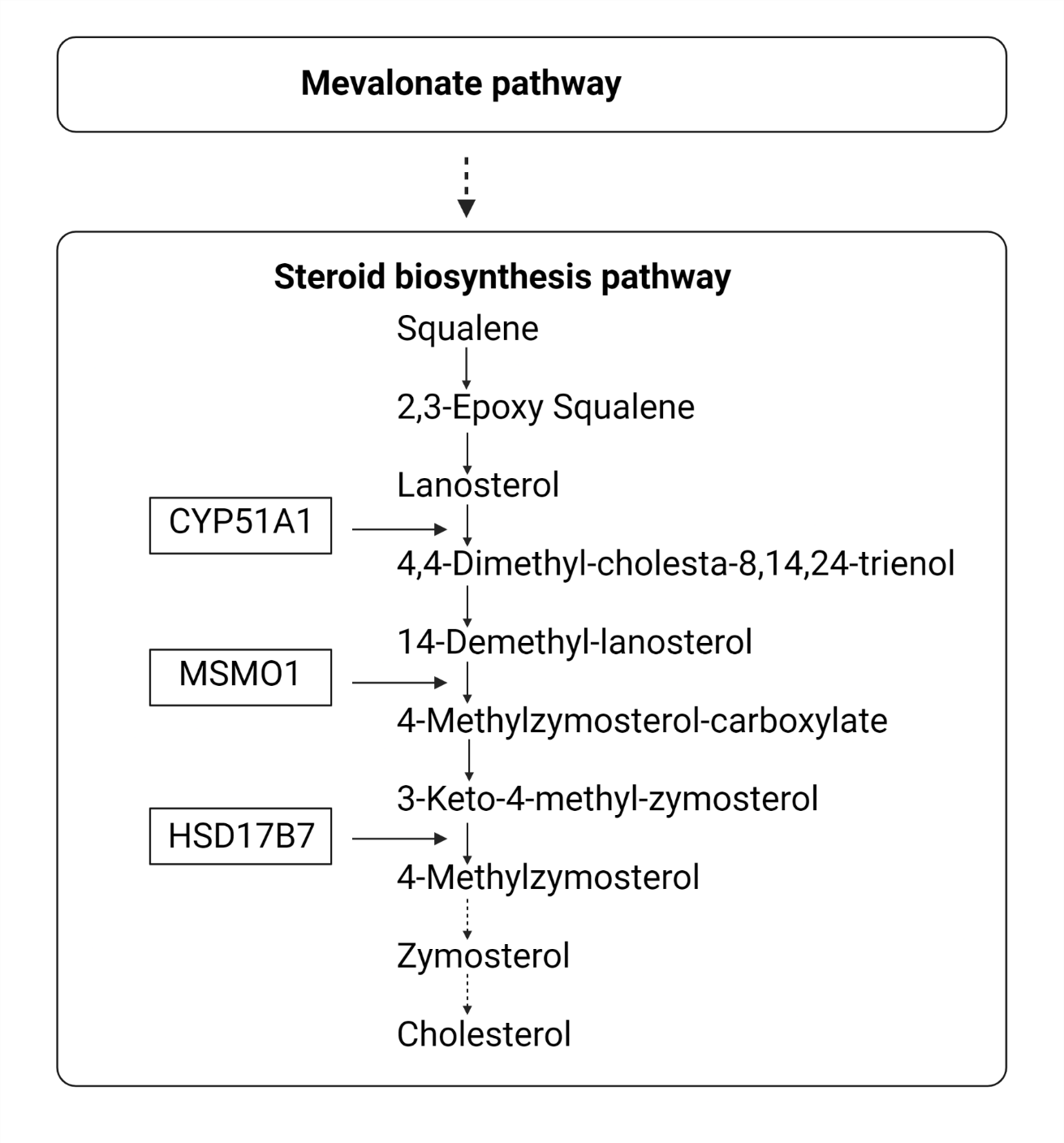


**Supplementary file 7: Pathway for cholesterol synthesis.** Cholesterol synthesis occurs via steroid biosynthesis pathway, which begins with mevalonate pathway. Three proteins identified to be significantly more enriched in luteal phase compared to follicular phase are marked: Cytochrome P450 family 51 subfamily A member 1 (CYP51A1), Methylsterol monooxygenase 1 (MSMO1) and Hydroxysteroid 17-beta dehydrogenase 7 (HSD17B7).
